# Decoupling the pleiotropic effects of *VRT-A2* during reproductive development enhances wheat grain length and weight

**DOI:** 10.1093/plcell/koaf024

**Published:** 2025-02-14

**Authors:** Jing Liu, Chaoqun Dong, Xiangqing Liu, Jinquan Guo, Lingling Chai, Weilong Guo, Zhongfu Ni, Qixin Sun, Jie Liu

**Affiliations:** State Key Laboratory of High-Efficiency Production of Wheat-Maize Double Cropping, Frontiers Science Center for Molecular Design Breeding, China Agricultural University, Beijing 100193, China; State Key Laboratory of High-Efficiency Production of Wheat-Maize Double Cropping, Frontiers Science Center for Molecular Design Breeding, China Agricultural University, Beijing 100193, China; State Key Laboratory of High-Efficiency Production of Wheat-Maize Double Cropping, Frontiers Science Center for Molecular Design Breeding, China Agricultural University, Beijing 100193, China; State Key Laboratory of High-Efficiency Production of Wheat-Maize Double Cropping, Frontiers Science Center for Molecular Design Breeding, China Agricultural University, Beijing 100193, China; State Key Laboratory of High-Efficiency Production of Wheat-Maize Double Cropping, Frontiers Science Center for Molecular Design Breeding, China Agricultural University, Beijing 100193, China; State Key Laboratory of High-Efficiency Production of Wheat-Maize Double Cropping, Frontiers Science Center for Molecular Design Breeding, China Agricultural University, Beijing 100193, China; State Key Laboratory of High-Efficiency Production of Wheat-Maize Double Cropping, Frontiers Science Center for Molecular Design Breeding, China Agricultural University, Beijing 100193, China; State Key Laboratory of High-Efficiency Production of Wheat-Maize Double Cropping, Frontiers Science Center for Molecular Design Breeding, China Agricultural University, Beijing 100193, China; State Key Laboratory of High-Efficiency Production of Wheat-Maize Double Cropping, Frontiers Science Center for Molecular Design Breeding, China Agricultural University, Beijing 100193, China

## Abstract

*VEGETATIVE TO REPRODUCTIVE TRANSITION 2* (*VRT-A2*) is a subspecies-forming gene that confers the long-glume and large-grain traits of tetraploid Polish wheat (*Triticum polonicum*; AABB) and hexaploid Xinjiang rice wheat (*T. petropavlovskyi*; AABBDD). Transcriptional activation of *VRT-A2* due to a natural sequence variation in its Intron-1 region significantly enhances grain weight but also causes some basal spikelets to fail to completely develop, thus decreasing grain number per spike and yield. This yield penalty has presented a challenge for the use of *VRT-A2* in breeding high-yield wheat. Here, we report the characterization of 2 regulatory modules that fine-tune *VRT-A2* expression in bread wheat (*T. aestivum*): (i) the APETALA2/Ethylene Responsive Factor (AP2/ERF)-type transcription factor MULTI-FLORET SPIKELET1 (TaMFS1) represses *VRT-A2* expression by recruiting a transcriptional corepressor and a histone deacetylase and (ii) the STRUCTURE-SPECIFIC RECOGNITION PROTEIN 1 (TaSSRP1) facilitates *VRT-A2* activation by assembling Mediator and further RNA polymerase II. Deleting *TaMFS1* triggered moderate upregulation of *VRT-A2* results in significantly increased grain weight without the yield penalty. Our study thus provides a feasible strategy for overcoming the tradeoffs of pleotropic genes by editing their upstream transcriptional regulators.

## Teaser

Deleting a gene that encodes a repressor of the subspecies-forming gene *VRT-A2* triggers its moderate upregulation, thereby enhancing grain weight without yield penalty in wheat.

## Introduction

Wheat is one of the most important staple food crops cultivated worldwide and provides up to 20% of the daily calorie intake by humans (http://faostat.fao.org). Continuously improving the yield of wheat is crucial for feeding the world's growing population. Wheat yield is a complex trait that is determined by 3 major components, i.e. spike number per unit area, grain number per spike (GNS), and thousand grain weight (TGW), among which GNS and TGW are strongly determined by the morphology of spike, the major reproductive organ of wheat.

Tetraploid Polish wheat (*Triticum polonicum*; AABB) and hexaploid Xinjiang rice wheat (*T. petropavlovskyi*; AABBDD) are 2 well-known subspecies in the genus *Triticum*, commonly characterized by their elongated glumes and large grains. These unique traits have led them to be valuable genetic resources for the improvement of the yielding traits, especially TGW, of modern wheat cultivars. Recently, several parallel studies have investigated the genetic mechanisms underlying the long-glume and large-grain traits of Polish wheat and Xinjiang rice wheat and revealed that these traits are conferred by the ectopic activation of the *SHORT VEGETATIVE PHASE* (*SVP*)-clade MADS-box gene *VRT-A2* (*VEGETATIVE TO REPRODUCTIVE TRANSITION 2*) in spikes and grains (Adamski et al. 2021; Liu et al. 2021; Xiao et al. 2021; Chai et al. 2022). These findings shed new light on a possible strategy to improve grain weight and yield of wheat by engineering *VRT-A2* expression.

However, it is noted that excessive activation of *VRT-A2* may also lead to strong and undesired pleiotropic effects, for example, increased number of rudimentary basal spikelets, decreased fertility and GNS, and the conversion of spikelets to leafy structures (Okamoto and Takumi 2013; Li et al. 2021; Liu et al. 2021; Backhaus et al. 2022). Due to the considerably complicated effects of *VRT-A2* in spike/spikelet development and grain weight determination, the utilization of this gene in wheat high-yield breeding practice has been largely restricted. Some transcription factors (TFs), such as the APETALA2/Ethylene Responsive Factor (AP2/ERF)-type MULTI-FLORET SPIKELET1 (TaMFS1), members of LATERAL ORGAN BOUNDARIES-DOMAIN, basic leucine zipper, B3, and GLABROUS1 enhancer-binding protein families, are proposed to be the upstream transcriptional regulators of *VRT-A2* (Adamski et al. 2021; Liu et al. 2021). Further characterization of the biological functions of these regulators may help to optimize the in vivo expression level of *VRT-A2* with reduced gene pleiotropies.

Our previous study has revealed that the GCC-box cis-elements located in the Intron-1 region of *VRT-A2* are potentially associated by the TF TaMFS1, which may contribute to the timely suppression of *VRT-A2* expression; conversely, the presence of intron-mediated enhancement (IME) motif(s) in the Intron-1 region of *VRT-A2* strongly facilitates *VRT-A2* activation (Liu et al. 2021). It is likely that these closely linked GCC-box and IME motifs in the Intron-1 of *VRT-A2* form a functional unit of “ON/OFF” molecular switch to tightly control the spatiotemporal expression of *VRT-A2*. Beyond *VRT-A2*, multiple plant genes have also been reported to be transcriptionally regulated via IME, i.e. their expression levels are strongly stimulated by their promoter-proximal introns. These genes include *Alcohol dehydrogenase-1* (*Adh1*), *Shrunken-1* (*Sh1*), and *Heat shock protein 82* (*Hsp82*) from maize (*Zea mays*); *SALT-RELATIVE PROTEIN* (*SalT*), *ACTIN 1* (*ACT1)*, and *TRIOSEPHOSPHATE ISOMERASE* (*TPI*) from rice (*Oryza sativa*); *Sucrose Synthase 3* (*Sus3*) from potato (*Solanum tuberosum*); and *TRYPTOPHAN BIOSYNTHESIS 1* (*TRP1*), *UBIQUITIN 10* (*UBQ10*), *PHOSPHORIBOSYLANTHRANILATE TRANSFERASE 1* (*PAT1*), *AGAMOUS* (*AG*), *MAGNESIUM/PROTON EXCHANGER 1* (*MHX*), and *PROFILIN 2* (*PRF2*) from *Arabidopsis thaliana* (Backhouse 1918; Callis et al. 1987; McElroy et al. 1990; Sinibaldi and Mettler 1992; Norris et al. 1993; Xu et al. 1994; Zhang et al. 1994; Fu et al. 1995; Snowden et al. 1996; Rethmeier et al. 1997; Rose and Last 1997; Sieburth and Meyerowitz 1997; Rose and Beliakoff 2000; Clancy and Hannah 2002; Rose 2002; Jeong et al. 2005; Akua and Shaul 2013). Although IME represents a general transcriptional regulation event in plant species, the regulatory protein candidates and the precise molecular mechanisms underlying the control of IME are largely unknown (Zalabák and Ikeda 2020).

RNA Polymerase II (RNA Pol II) is a major part of preinitiation complex (PIC) that is globally required for transcriptional activation of all protein-coding genes. Mechanistically, RNA Pol II recruits the multisubunit Mediator by its C-terminal domain (CTD), which further enables the recruitment of other specific TFs for gene activation (Roeder 1996; Allen and Taatjes 2015; Soutourina 2018; Zhai and Li 2019; Chen et al. 2022a). Despite this classic model of PIC assembly for transcriptional activation, several recent studies in mammalian species highlight an essential role of phase-separated multimolecular assemblies in compartmentalizing the transcriptional events in the nucleus and concentrating multiple transcriptional activators for more effective gene activation (Hnisz et al. 2017; Boija et al. 2018; Sabari et al. 2018). According to this model, the Mediator subunits, for example MED1, carry intrinsically disordered regions and can form phase-separated condensates with other transcriptional activators, including Bromodomain-containing protein 4, octamer-binding transcription factor 4 and general control nonderepressible 4, to facilitate gene activation (Boija et al. 2018; Sabari et al. 2018). However, until present, the potential roles of these transcriptional cofactors in controlling gene transcription in plant species, especially in wheat (*Triticum* spp.), have not been well documented.

In this work, we aim to elucidate the molecular mechanisms underlying the sophisticated transcriptional control of *VRT-A2* in wheat. First, we provide in vivo genetic evidence to confirm that TaMFS1 represses *VRT-A2* expression. In addition, we identify a transcriptional regulator, the STRUCTURE-SPECIFIC RECOGNITION PROTEIN 1 (TaSSRP1), that directly associates with the IME motifs in the Intron-1 region of *VRT-A2* to facilitate the transcriptional activation of *VRT-A2*. In addition, TaSSRP1 interacts with Mediator and RNA Pol II and colocalizes with these components in the nucleus. These findings represent an exquisitely designed “repressor–activator” pair for the control of *VRT-A2* transcription. By tipping this TaMFS1–TaSSRP1 balance toward increased TaSSRP1 activity through the knockout of *TaMFS1*, we enable a moderate upregulation of *VRT-A2* expression and significantly increased grain yield per spike in wheat.

## Results

### TaMFS1 suppresses glume and grain elongation in wheat

**Figure 1.**
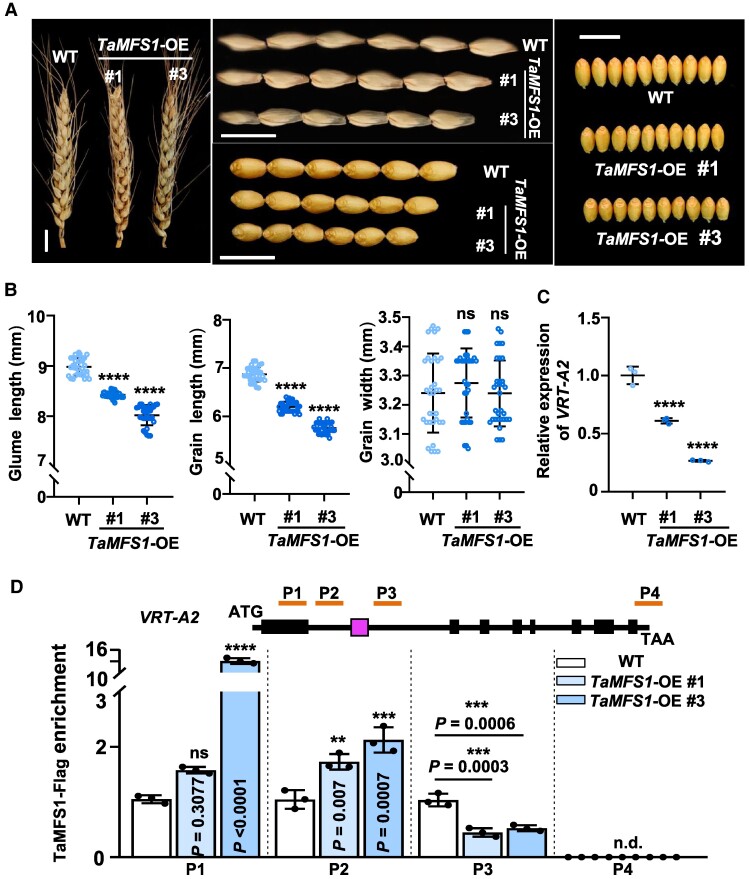
*TaMFS1*-OE suppresses glume and grain elongation as well as *VRT-A2* expression. **A)** Spikes, glumes, and grains of WT Fielder and *TaMFS1*-OE transgenic lines (T_2_ generation, #1 and #3, at the genetic background of Fielder). Plants used for phenotypic analyses and data collection were planted in the pots and grown in a growth chamber. Scale bars, 1 cm. Images were digitally extracted for comparison. **B)** Statistical analyses of the phenotypes shown in **A)**. Data are means ± Sd (*n* = 33 pot-grown plants from 3 independent biological replications). **C)** RT-qPCR assay showing the expression levels of *VRT-A2* in the spikes of WT and *TaMFS1*-OE lines. For RT-qPCR analysis, the leaf samples of WT and *TaMFS1*-OE plants grown in a growth chamber were collected for analysis when the spikes were 0.5 cm in length. The relative expression level of *VRT-A2* in WT was set to 1. The *Actin* gene was used as an internal control. Data are shown as means ± Sd (*n* = 3 independent biological replications). **D)** ChIP-PCR assay showing that TaMFS1-Flag associates with the Intron-1 region of *VRT-A2* in vivo. The leaves of WT and *TaMFS1*-OE (#1 and #3) were collected for the ChIP-PCR assay when their spikes were about 0.5 cm in length. A schematic representation showing the gene structure of *VRT-A2*, and P1 to P4 represent the 4 amplicons detected in the ChIP-PCR assay. The box between amplicons P2 and P3 indicates the position of the 560-bp fragment located in the Intron-1 of the Fielder-derived *VRT-A2a* allele. Immunoprecipitation was performed with anti-Flag antibody, and data are means ± Sd (*n* = 3 independent biological replications). In **B)**, **C)**, and **D)**, ****P < 0.0001, ***P < 0.001, **P < 0.01 (1-way ANOVA followed by Dunnett's tests); ns, no significant difference; n.d., not detected.

Our previous in vitro data have shown that TaMFS1 represses *VRT-A2* transcription through direct association with the Intron-1 region of *VRT-A2* (Liu et al. 2021). However, the in vivo effects of TaMFS1 on *VRT-A2* expression and the potential biological roles of *TaMFS1* in regulating glume/spike morphology and grain traits remain to be elucidated. Here, we generated 2 independent *TaMFS1*-overexpression transgenic lines (*TaMFS1*-OE, #1 and #3; in this study, the A subgenome-derived *TaMFS1-A* was used as a representative for functional analyses) by introducing an expression cassette containing the intact ORF of *TaMFS1* driven by the *Ubiquitin* promoter into the hexaploid spring wheat cultivar Fielder. Immunoblotting assay confirmed that the Flag-tagged TaMFS1 proteins were highly accumulated in these *TaMFS1*-OE lines (Supplementary Fig. S1A). Phenotypic analysis revealed that these transgenic lines showed significantly reduced glume length and grain length than the wild-type (WT) Fielder plants, while the grain width of these lines was similar with that in WT (Fig. 1, A and B), contrasting to the effect of *VRT-A2* in promoting glume and grain elongation. Indeed, when we carried out reverse transcription quantitative PCR (RT-qPCR) assay, we confirmed that the in vivo transcript levels of *VRT-A2* were significantly lower in *TaMFS1*-OE (#1 and #3) lines than in WT (Fig. 1C; Supplementary Fig. S1B). In support of this, chromatin immunoprecipitation (ChIP) PCR assay by using the leaf samples of *TaMFS1*-OE plants revealed that TaMFS1 proteins could directly bind to the Intron-1 region of *VRT-A2* gene (Fig. 1D). These findings well support the previous finding that TaMFS1 acts as an upstream transcriptional repressor of *VRT-A2* (Liu et al. 2021). In addition, RT-qPCR assays revealed that the expression of *VRT-B2* and *VRT-D2*, the 2 homoeologous genes of *VRT-A2*, was also significantly repressed by *TaMFS1*-OE in the 0.5-cm spike tissues (Supplementary Fig. S1C), suggesting highly conserved effect of TaMFS1 in repressing different homoeologs of *VRT2* gene. We also determined the expression of *SVP-A1*, a glume elongation gene that has similar effect with *VRT-A2* to confer elongated glume and floral organ in *T. ispahanicum* (Chen et al. 2022b). However, *SVP-A1* expression was not changed by the *TaMFS1*-OE (Supplementary Fig. S1D), suggesting a specific regulatory role of TaMFS1 on *VRT2* expression. Taken together, we conclude that *TaMFS1* overaccumulation in wheat suppresses glume and grain elongation, which is consistent with its biological effect in associating with *VRT2* gene for transcriptional repression (Liu et al. 2021).

### TaMFS1 recruits TOPLESS and TaHD2A for transcriptional repression

**Figure 2.**
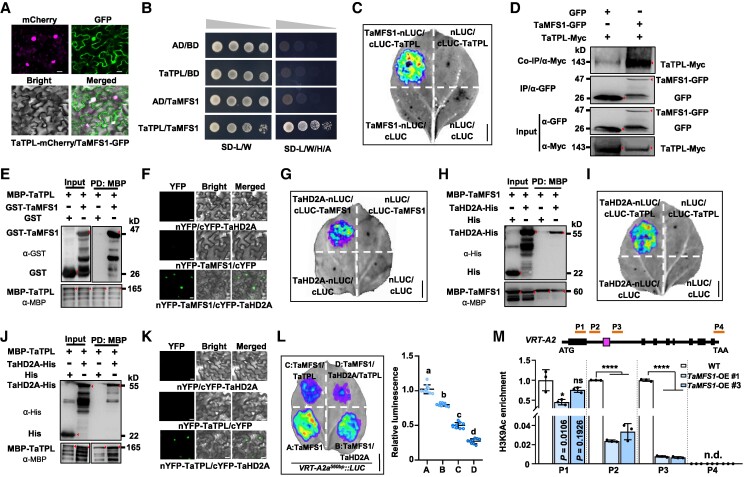
TaMFS1 interacts with TaTPL and TaHD2A and represses the expression of *VRT-A2*. **A)** Colocalization of TaTPL-mCherry and TaMFS1-GFP in *N. benthamiana* leaf epidermal cells. **B)** Y2H showing the interaction between TaMFS1 and TaTPL. The transformed yeast cells were grown on SD medium lacking Leu and Trp (SD-L/W) and selected on SD lacking Leu, Trp, His, and Ade (SD-L/W/H/A). **C)** LCI assay confirming the TaMFS1–TaTPL interaction. **D)** Co-IP assay showing the TaMFS1–TaTPL interaction. The anti-GFP affinity beads were used for IP, and anti-GFP and anti-Myc antibodies were used for immunoblotting. Total proteins before IP were employed as the input. **E)** Pull-down assay validating the in vitro TaMFS1–TaTPL interaction. GST was used as a negative control. Blots were detected with the anti-GST and anti-MBP antibodies. **F)** BiFC showing the interaction between TaMFS1 and TaHD2A. LCI **G)** and pull-down **H)** assays confirming the TaMFS1–TaHD2A interaction. His EV was used as a negative control. **I)** LCI, **J)** pull-down, and **K)** BiFC assays showing the TaTPL–TaHD2A interaction. **L)** Expression of TaTPL and/or TaHD2A facilitates the TaMFS1-mediated transcriptional repression of the reporter *VRT-A2a^560bp^::LUC*. Data are means ± Sd (*n* = 9 independent leaf samples). Different lowercase letters indicate significant differences based on 1-way ANOVA with Tukey's multiple comparisons test (*P* < 0.05). **M)** ChIP-PCR assay showing the levels of histone H3 acetylation (H3K9Ac) modification on the *VRT-A2* gene region in WT and *TaMFS1*-OE (#1 and #3) plants. A schematic representation showing the gene structure of the Fielder-derived *VRT-A2* (the *VRT-A2a* allele), and P1 to P4 represent the 4 amplicons detected in the ChIP-PCR assay. The box between amplicons P2 and P3 indicates the position of the 560-bp fragment in the Intron-1 of *VRT-A2a*. Data are means ± Sd (*n* = 3 biologically independent replications). ****Shows significant difference between WT and *TaMFS1*-OE at *P* < 0.0001 level; *P < 0.05 (1-way ANOVA followed by Dunnett's); ns, no significant difference; n.d., not detected. In **A)**, **F)**, and **K)**, scale bars are 20 *μ*m. In **C)**, **G)**, **I)**, and **L)**, scale bars are 1 cm. The images in **C)**, **G)**, **I)**, and **L)** were digitally extracted for comparison. In **D)**, **E)**, **H)**, and **J)**, red triangles indicate the target protein bands. cYFP, C-terminal part of YFP; nYFP, N-terminal part of YFP; AD, GAL4 activation domain; BD, GAL4 DNA-binding domain.

To understand the transcriptional repression effect of TaMFS1, we made efforts to identify the TaMFS1 interaction proteins. Protein sequence analysis revealed that TaMFS1 belongs to the AP2/ERF family TF that harbors a linear sequence, Asp-Leu-Asn-Glu-Pro-Pro (DLNEPPF) (Liu et al. 2021), matching the highly conserved ethylene-responsive element binding factor–associated amphiphilic repression (EAR) motif DLNxxP (Kagale and Rozwadowski 2010). This hallmark of transcriptional repression motif prompted us to test whether TaMFS1 may directly interact with TOPLESS (TPL), the corepressor that is normally recruited by TFs for transcriptional repression through the EAR motifs (Szemenyei et al. 2008; Pauwels et al. 2010). Indeed, as shown in subcellular localization assay in *Nicotiana benthamiana* leaf epidermal cells, the green fluorescence of TaMFS1-GFP was partially merged with the red fluorescence of TaTPL-mCherry (*TaTPL-A1* homoeologous gene derived from A subgenome was used for functional analyses) in the nucleus (Fig. 2A), and the TaMFS1–TaTPL interaction was also validated by yeast 2-hybrid (Y2H), firefly luciferase (LUC) complementation imaging (LCI), co-immunoprecipitation (Co-IP), and pull-down assays (Fig. 2, B to E).

Screening for TaMFS1 interactors by a Y2H also characterized TraesCS1A02G445700 as a candidate (Supplementary Data Set 1). Based on protein sequence alignment, we showed that TraesCS1A02G445700 is phylogenetically close to the Arabidopsis HD2 subfamily (Supplementary Fig. S2) members of histone deacetylases (HDACs), which are important chromatin regulators that mediate histone lysine deacetylation and affect gene expression (Cui et al. 2023). Therefore, we named TraesCS1A02G445700 as TaHD2A-A1 (derived from A subgenome; hereafter we use TaHD2A for abbreviation). The TaMFS1–TaHD2A interaction was validated by the in vivo bimolecular fluorescence complementation (BiFC) and LCI assays and the in vitro pull-down assay (Fig. 2, F to H). In addition to TaHD2A, we also selected its homologous proteins TaHDA1-A (encoded by TraesCS7A02G365600) and TaHDA6-A (encoded by TraesCS6A02G181100) that belong to the RPD3/HDA1 family of HDACs (Supplementary Fig. S2) for analysis and determined their potential interactions with TaMFS1. Nevertheless, under our current experimental conditions, no detectable interaction signal was observed between TaHDA1-A and TaMFS1 or between TaHDA6-A and TaMFS1 (Supplementary Fig. S3).

Given that TaMFS1 interacts with both TaTPL and TaHD2A, we wondered if TaTPL and TaHD2A may also interact with each other. As expected, LCI and pull-down assays revealed strong interaction signals between TaHD2A and TaTPL (Fig. 2, I and J). Furthermore, BiFC assay supported that the interaction of TaHD2A with TaTPL occurs exclusively in the nucleus (Fig. 2K). Consistent with these physical interactions, subcellular localization assays in *N. benthamiana* revealed partially merged localization patterns of TaMFS1, TaTPL, and TaHD2A in the nucleus (Supplementary Fig. S4), and RT-qPCR assay revealed similar expression patterns of *TaMFS1*, *TaTPL*, and *TaHD2A* genes in different wheat tissues (Supplementary Fig. S5). Importantly, transcriptional activation activity assay confirmed that both the presence of TaHD2A and TaTPL proteins could significantly enhance the transcriptional repression activity of TaMFS1 on the regulation of *VRT-A2* expression (Fig. 2L), indicating putatively additive effects among these 3 proteins in regulating a common downstream gene.

We further explored potential effects of TaMFS1 in arranging local histone acetylation modification patterns at the TaMFS1-targeted *VRT-A2* gene locus. In this assay, we collected the leaf samples of the *TaMFS1-*OE transgenic plants and conducted ChIP-PCR assay by using the anti-histone H3 acetylation (anti-H3K9Ac) and anti-histone H3 trimethylation of lysine 27 (anti-H3K27me3) antibodies. Consistent with downregulated expression levels of *VRT-A2* in *TaMFS1-*OE lines (Fig. 1C), the H3K9ac levels at the *VRT-A2* gene locus were also significantly lower in these lines than in WT (Fig. 2M). These findings indicate a potential effect of TaMFS1 in changing the histone acetylation levels at the *VRT-A2* gene region. We also determined the H3K27me3 modification levels at the *VRT-A2* gene region but found contrasting modification patterns in the lines #1 and #3 of *TaMFS1-*OE (Supplementary Fig. S6), suggesting that TaMFS1 might have no or negligible effect on H3K27me3 modification.

### TaSSRP1 is an upstream activator of *VRT-A2*

Although previous studies in *A. thaliana*, rice (*O. sativa*) and Polish wheat (*T. polonicum*) have confirmed the critical role of IME super-enhancing motifs in facilitating gene activation (Rose et al. 2008; Morello et al. 2011; Liu et al. 2021), to date, how these IME elements contribute to gene activation in plant species remains largely unknown. In human embryonic stem cells, some key master TFs, such as SOX2, SOX9 and SOX10, have been reported to control super-enhancer assembly and gene activation (Werner et al. 2007; Sekido and Lovell-Badge 2008; Bhattarai et al. 2022), which prompted us to test whether the plant homologs of these master TFs may be conservatively involved in the IME-mediated gene activation in plant species. Phylogenetic analysis revealed that the SOX family members were closely related to the STRUCTURE-SPECIFIC RECOGNITION PROTEIN 1 (SSRP1) derived from Arabidopsis and the grass family Poaceae (Supplementary Fig. S7A). We analyzed the spatial expression pattern of *SSRP1* gene in wheat (*TaSSRP1*), and found that this gene is nontissue specifically expressed in all tested tissue samples, with relatively higher expression level in the 1-cm spike than in other tested tissues (Supplementary Fig. S7B).

**Figure 3.**
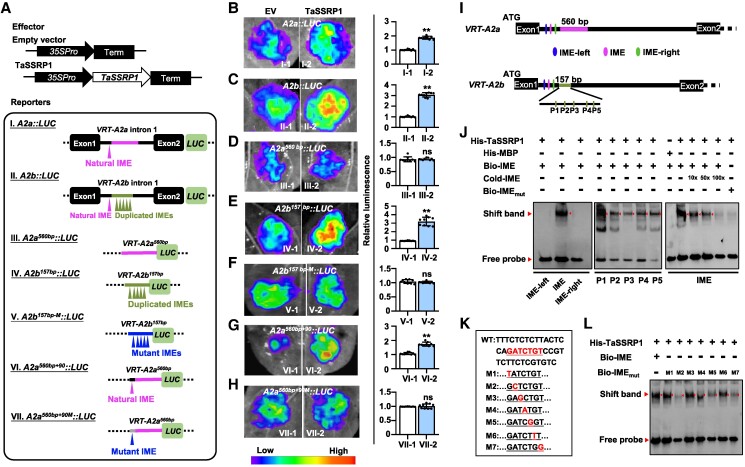
TaSSRP1 regulates *VRT-A2* expression through the association with the IME motifs. **A)** Schematic diagrams representing the effectors and reporters used in the study. Lines in magenta color indicate the 560-bp Intron-1 fragment in *VRT-A2a* allele, and lines in olive illustrate the 157-bp Intron-1 fragment found in *VRT-A2b* allele. **B** to **H)** Transcriptional activation activity assays in *N. benthamiana* leaves using the effectors including EV and TaSSRP1 and the reporters including I to VII shown in **A)**. The colored scale bar indicates the relative luminescence intensity. Data are means ± Sd (*n* = 9 biologically independent samples). **Statistically significant difference between EV and TaSSRP1-expressing samples at *P* < 0.01 level (2-tailed Student's *t*-test); ns, no significant difference. In **B)** to **H)**, *P* = 1.40 × 10^−13^, 2.55 × 10^−10^, 0.8969, 7.77 × 10^−7^, 0.4063, 9.2 × 10^−10^, and 0.6344, respectively. **I)** Schematic representations of the Exon-1, Intron-1, and Exon-2 regions of *VRT-A2a* and *VRT-A2b* alleles. IME, a probe containing the natural IME motif shared by *VRT-A2a* and *VRT-A2b*; IME-left and IME-right: the probes representing the left and right flanking sequences of the natural IME motif, respectively; P1 to P5, 5 probes containing the 5 IME motifs in the 157-bp sequence specific in *VRT-A2b*, respectively. The 560-bp and 157-bp fragments were highlighted separately in the Intron-1 regions of *VRT-A2a* and *VRT-A2b*. **J)** EMSA showing that His-TaSSRP1 recombinant protein can specifically bind to the biotin-labeled probes (IME and P1-P5) containing the intact IME motifs. The red triangles represent the DNA–protein complexes. Cold-IME represents the unlabeled IME probe used as competitor. His-MBP was used as a negative control. **K)** Design of probes containing single nucleotide mutations in the IME motif. WT shows the probe sequence containing the intact IME motif, while M1 to M7 show the probes containing different point mutations within the IME motif (highlighted in red). **L)** EMSA assay using His-TaSSRP1 and probes as shown in **K)**. The red triangles represent the DNA–protein complex.

To determine putative effects of TaSSRP1 in facilitating *VRT-A2* gene activation, we carried out transient transcriptional activation activity assay. In such assay, we employed the previously constructed *A2a::LUC* and *A2b::LUC* reporters, in which the Exon-1/Intron-1/Exon-2 fragments of the *VRT-A2a* and *VRT-A2b* alleles were separately fused with the *LUC* reporter gene (Fig. 3A; Supplementary Data Set 2) (Liu et al. 2021). When coexpressing these reporters with TaSSRP1 (the A subgenome-derived TaSSRP1-A was used as a representative for analysis) or the negative control empty vector (EV) in the leaf epidermal cells of *N. benthamiana*, we found that TaSSRP1 could significantly activate the expression of both *A2a::LUC* and *A2b::LUC* reporter genes (Fig. 3, B and C), supporting the notion that TaSSRP1 is a common transcriptional activator for the regulation of *VRT-A2a* and *VRT-A2b*. To test whether this TaSSRP1-mediated *VRT-A2a/VRT-A2b* activation is related to the IME motifs in the Intron-1 regions, we generated 2 reporter systems, including *A2a^560bp^::LUC* that carries the *VRT-A2a*-specific 560-bp sequence with no IME motif and *A2b^157bp^::LUC* that harbors the *VRT-A2b*-specific 157-bp sequence with duplicated IME motifs (Fig. 3A, III and IV). Experimental data confirmed that TaSSRP1 specifically activated *A2b^157bp^::LUC* while had no significant influence on the expression of *A2a^560bp^::LUC* (Fig. 3, D and E). Moreover, when we mutated the duplicated IME motifs in the *A2b^157bp^::LUC* reporter to generate a *A2b^157bp-M^::LUC* reporter (Fig. 3, A and V), the TaSSRP1-mediated activation of the *LUC* reporter was completely abolished (Fig. 3F). In a parallel analysis, we fused the 560-bp *VRT-A2a*-specific Intron-1 sequence together with its left flanking 90-bp sequence harboring a natural IME motif to generate a *A2a^560bp + 90^::LUC* reporter (Fig. 3A, VI). Using this reporter, we observed strong upregulation of LUC activity by the expression of TaSSRP1 (Fig. 3G). Nevertheless, mutation in the natural IME motif in this *A2a^560bp + 90^::LUC* reporter, represented by *A2a^560bp + 90M^::LUC* (Fig. 3A, VII), completely abolished the TaSSRP1-mediated activation of LUC activity (Fig. 3H). Together, these results strongly support that the IME motifs located in the Intron-1 regions of *VRT-A2a* and *VRT-A2b* are essentially required for the TaSSRP1-mediated activation of *VRT-A2* expression.

Based on the above findings, we further assumed that TaSSRP1 may directly associate with the IME motif. To test this, we performed electrophoretic mobility shift assay (EMSA) by using the *Escherichia coli* BL21 (DE3)–expressed His-TaSSRP1 protein and the biotin-conjugated probes designed according to the Intron-1 sequences of *VRT-A2a* and *VRT-A2b* with or without IME motifs (Supplementary Fig. S8). As expected, we demonstrated specific binding of His-TaSSRP1 to the probes containing intact IME motifs, represented by the probes of Bio-IME and Bio-P1 to Bio-P5; whereas no binding signal was observed between His-TaSSRP1 and the probes without IME motif, these include Bio-IME-left and Bio-IME-right designed according to the left and right flanking sequences of the natural IME motif (Fig. 3, I and J). To further test which nucleotide(s) within the IME motif (i.e. GATCTGT) may be essential for TaSSRP1 binding, we introduced single nucleotide mutations in the IME motif (Fig. 3K). EMSA results revealed that the 2nd 4th, 5^th^, and 7th nucleotides within this GATCTGT sequence are crucial nucleotides that their mutations would largely attenuate TaSSRP1 binding (Fig. 3L). Taken together, our findings support a notion that TaSSRP1 may facilitate *VRT-A2* expression through direct association with the IME motifs within the Intron-1 regions of *VRT-A2a* and *VRT-A2b*.

### TaSSRP1 facilitates *VRT2* activation and glume/grain elongation

**Figure 4.**
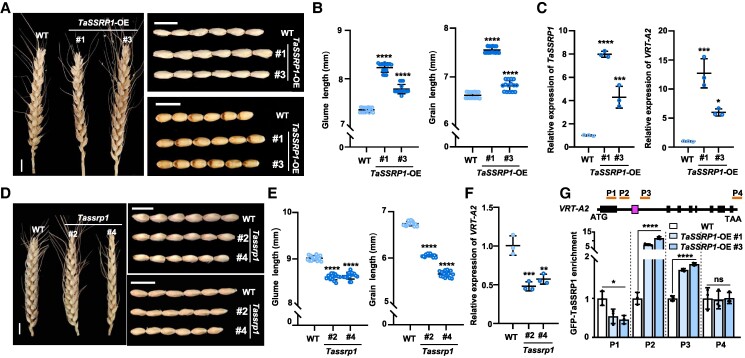
TaSSRP1 facilitates glume and grain elongation and promotes *VRT-A2* expression. **A)** Spikes, glumes, and grains of WT and *TaSSRP1*-OE transgenic plants. Images were digitally extracted for comparison. **B)** Statistical analyses of the phenotypes as shown in **A)**. Data are means ± Sd (*n* = 15 pot-grown plants from 3 independent biological replications). **C)** RT-qPCR revealing significantly higher expression levels of *TaSSRP1* and *VRT-A2* in the spike tissues of *TaSSRP1*-OE transgenic plants relative to that in WT. Data are means ± Sd (*n* = 3 independent biological replications). **D)** Spikes, glumes, and grains of WT and *Tassrp1* mutant plants. Images were digitally extracted for comparison. **E)** Statistical analyses of the phenotypes as shown in **D)**. Data are means ± Sd (*n* = 15 pots from 3 independent biological replications). **F)** RT-qPCR assay showing significantly lower expression levels of *VRT-A2* in the spikes of *Tassrp1* mutant plants relative to that in WT. Data are means ± Sd (*n* = 3 independent biological replications). **G)** ChIP-PCR assay confirming the enrichment of TaSSRP1-GFP proteins on the gene region of *VRT-A2* in *TaSSRP1*-OE. The 3- to 4-cm spikes of WT and *TaSSRP1*-OE were collected for the ChIP-PCR assay. The immunoprecipitation was conducted by using the anti-GFP antibody. A schematic representation showing the gene structure of the Fielder-derived *VRT-A2* (the *VRT-A2a* allele), and P1 to P4 represent the 4 amplicons detected in the ChIP-PCR assay. The box between amplicons P2 and P3 indicates the position of the 560-bp fragment in the Intron-1 of *VRT-A2a*. Data are means ± Sd (*n* = 3 biologically independent replications). Plants used for phenotypic analyses and data collection were planted in the pots and grown in a growth chamber. Scale bars in **A)** and **D)** are 1 cm. In **B)**, **C)**, **E)**, **F)**, and **G)**, asterisks represent significant differences between WT and *TaSSRP1*-OE based on 1-way ANOVA followed by Dunnett's tests (****P < 0.0001; ***P < 0.001; **P < 0.01; *P < 0.05). ns, no significant difference.

Since TaSSRP1 activates *VRT-A2* expression, next we aimed to evaluate the biological significance of TaSSRP1 in regulating the sizes of glumes and grains in wheat. To this end, we generated *TaSSRP1* overexpression lines (*TaSSRP1*-OE, #1 and #3; in which *TaSSRP1* expression was driven by the *Ubiquitin* promoter) under the genetic background of c.v. Fielder (Fig. 4A). In agreement with the elevated expression levels of *TaSSRP1* in *TaSSRP1-*OE transgenic plants, the expression levels of *VRT-A2*, *VRT-B2*, and *VRT-D2* were all upregulated relative to that in WT (Fig. 4C; Supplementary Fig. S9C), confirming the TaSSRP1-mediated activation of *VRT2* expression in vivo. Phenotypic analysis demonstrated that the 2 independent lines of *TaSSRP1-*OE (#1 and #3) all exhibited longer glumes and grains than WT (Fig. 4B). Notably, the grain width was not affected by the *TaSSRP1*-OE (Supplementary Fig. S9A and B), suggesting a specific effect of *TaSSRP1* on grain elongation, which resembles the effect of *VRT-A2*. We also generated *Tassrp1* mutant lines (#2 and #4) via clustered regularly interspaced short palindromic repeat (CRISPR)/Cas9 gene editing strategy, in which 2 homoeologs of *TaSSRP1* from A and D subgenomes were simultaneously knocked out by gene editing (Supplementary Fig. S10). Contrasting to the long-glume and grain phenotypes of *TaSSRP1*-OE, the *Tassrp1* mutant plants showed significantly reduced glume and grain lengths when compared with WT (Fig. 4, D and E), while their grain widths were similar with WT (Supplementary Fig. S10B and C). In addition, *Tassrp1* conferred lower *VRT2* expression than WT (Fig. 4F; Supplementary Fig. S10D).

Using the spike samples of the *TaSSRP1-*OE transgenic plants, we conducted ChIP-PCR assay. Our data showed significant enrichment of TaSSRP1 proteins on the flanking regions of the IME motifs located in the Intron-1 of *VRT-A2* (Fig. 4G). Consistent with these findings, TaSSRP1 could preferentially bind to the lysine 9-acetylated histone H3 (H3K9ac) but not the lysine 27-trimethylated histone H3 (H3K27me3; Supplementary Fig. S11A), suggesting that TaSSRP1 is a histone binding protein. ChIP-PCR assay revealed that the *TaSSRP1*-OE plants conferred significantly higher H3K9ac modification levels across the TaSSRP1 association sites on the *VRT-A2* gene locus than WT, while showed lower H3K27me3 modification levels at these regions than WT (Supplementary Fig. S11B). These findings indicate a potential role of TaSSRP1 in affecting histone modification spanning *VRT-A2* gene region.

In summary, our genetic evidence supports that TaSSRP1 facilitates *VRT2* expression in vivo, which defines TaSSRP1 as a positive regulator for glume and grain elongation in wheat.

### TaSSRP1 interacts with TaMED6 to recruit RNA Polymerase II

**Figure 5.**
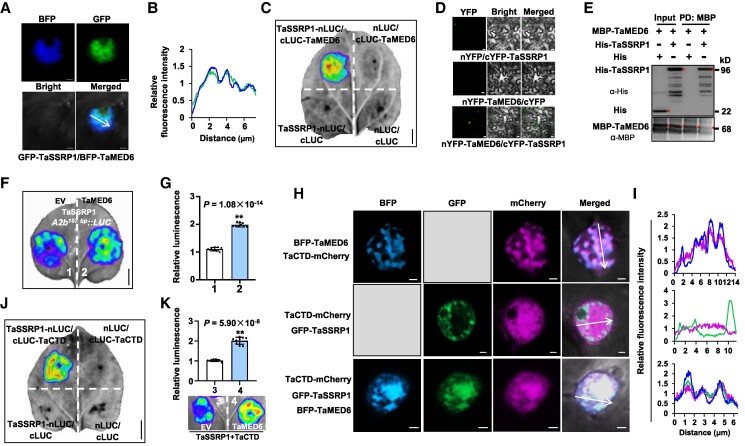
TaSSRP1 interacts with the mediator subunit TaMED6 and RNA Polymerase II. **A** and **B)** Subcellular colocalization pattern of GFP-TaSSRP1 with BFP-TaMED6 in *N. benthamiana* leaf cells. Scale bars, 2 *μ*m. A representative cell image was shown in **A)**, and relative fluorescence intensities of GFP-SSRP1 (green) and BFP-TaMED6 (blue) signals across the cell nucleus were shown in **B)**. The relative intensities were calculated by the Zen software on a Zeiss LSM 880 confocal microscope. Interaction between TaSSRP1 and TaMED6 confirmed by LCI **C)**, BiFC **D)**, and pull-down **E)** assays. Scale bars in **D)**, 20 *μ*m. Red triangles in **E)** indicate the target protein bands. **F** and **G)** Transient transcriptional activation assay showing that the presence of TaMED6 significantly facilitates the transcriptional activation activity of TaSSRP1 in activating *A2b^157bp^::LUC* reporter. A representative image was shown in **F)**, and statistical analysis of relative luminescence was given in **G)**. **H** and **I)** Subcellular localization patterns of BFP-TaMED6, GFP-TaSSRP1, and mCherry-TaCTD (the mCherry-tagged CTD of wheat RNA Polymerase II). Scale bars, 2 *μ*m. The gray color filled boxes illustrate that the corresponding channels were not detected. The representative cells were shown in **H)**, and relative fluorescence intensities of BFP-TaMED6, GFP-TaSSRP1, and mCherry-TaCTD signals across the cell nucleus were shown in **I)**. **J)** LCI assay revealing the interaction between TaSSRP1 and TaCTD. **K)** LCI assay showing that the interaction signal between TaSSRP1 and TaCTD was enhanced by the expression of TaMED6. In **G)** and **K)**, data are means ± Sd (*n* = 9 biologically independent samples), and asterisks (**) represent significant difference at *P* < 0.01 (2-tailed Student's *t*-test). In **C)**, **F)**, and **J)**, scale bars are 1 cm. The images of **C)**, **F)**, and **J)** were digitally extracted for comparison.

Considering that TaSSRP1 mediates the transcriptional activation of *VRT-A2*, we further asked whether this TaSSRP1-triggered gene transcription is functionally related to the Mediator complex. To this end, we selected TaMED6, TaMED8, TaMED4, and TaMED25 that are separately located in the head (TaMED6 and TaMED8), middle (TaMED4), and other unassigned parts (TaMED25) of the Mediator complex (Mao et al. 2019; Zhai and Li 2019), for further analysis. Subcellular localization assay showed that the GFP-tagged TaSSRP1 exhibited the punctate localization in the nucleus (Supplementary Fig. S12). Although all tested Mediator subunits, including TaMED4, TaMED6, TaMED8, and TaMED25 (here we employed the B subgenome-derived TaMED4-B1, the A subgenome-derived TaMED6-A1, the A subgenome-derived TaMED8-A1, and the D subgenome-derived TaMED25-D1 for functional analyses), formed puncta or droplets in the nucleus, their subcellular localization patterns were highly diversified regarding the droplet structure, number, and size (Supplementary Fig. S12). By performing colocalization assay, we confirmed that only BFP-TaMED6 (tagged with blue fluorescent protein), but not other Mediator subunits, could partly colocalize with GFP-TaSSRP1 (Fig. 5, A and B; Supplementary Figs. S13 and S14). In addition, *TaMED6* gene also showed higher expression levels in spikes at jointing stage than in other tissues (Supplementary Fig. S15), resembling that of *TaSSRP1* (Supplementary Fig. S7B). These observations indicate that TaSSRP1 and TaMED6 are likely spatially and functionally related. The direct interaction between TaSSRP1 and TaMED6 was further confirmed by the LCI and BiFC assays in plant cells and the in vitro pull-down assay (Fig. 5, C to E). Importantly, coexpression of TaMED6 with TaSSRP1 conferred stronger transcriptional activation activity than single expression of TaSSRP1 in facilitating the expression of *VRT-A2* (Fig. 5, F and G), indicating an additive effect between TaSSRP1 and TaMED6 in regulating *VRT-A2*.

Mediator complex mediates gene transcriptional activation through the recruitment of RNA Pol II (Conaway and Conaway 2011; Whyte et al. 2013; Zhai and Li 2019), we next asked whether the TaSSRP1–TaMED6 interaction may potentially bridge TaSSRP1–RNA Pol II association. To test this, we coexpressed GFP-TaSSRP1 with mCherry-TaCTD (the B subgenome-derived CTD of wheat RNA Pol II tagged with mCherry) or BFP-TaMED6 or both of them. In mCherry-TaCTD and BFP-TaMED6 coexpressed cells, overlapping punctate signals were observed (Fig. 5, H and I; Supplementary Fig. S14), indicating that RNA Pol II could be effectively recruited by TaMED6. When GFP-TaSSRP1 was coexpressed with mCherry-TaCTD in *N. benthamiana* leaf cells, we detected no or very weak colocalization of the punctate signal of GFP-TaSSRP1 with mCherry-TaCTD (Fig. 5, H and I; Supplementary Fig. S14), suggesting that TaSSRP1 and RNA Pol II themselves are likely not spatially associated with each other. However, upon the coexpression of the 3 proteins including GFP-TaSSRP1, BFP-TaMED6, and mCherry-TaCTD, the fluorescence of mCherry-TaCTD could be partially merged with the puncta of GFP-TaSSRP1 in the nucleus (Fig. 5, H and I; Supplementary Fig. S14). In support of this, we also coexpressed BFP-TaMED6 with TaSSRP1-nLUC and cLUC-TaCTD in *N. benthamiana* and found that the presence of BFP-TaMED6 significantly enhanced the interaction between TaSSRP1-nLUC and cLUC-TaCTD when compared with the BFP negative control sample (Fig. 5, J and K). Taken together, our findings confirm that in live plant cells, TaSSRP1 can interact with TaMED6, while this interaction may further bridge the spatial association of TaSSRP1 with RNA Pol II.

### Deleting *TaMFS1* enables an improved yield performance

**Figure 6.**
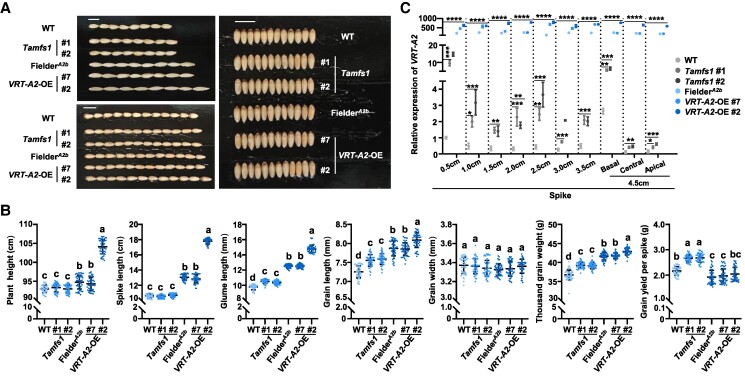
Deleting *TaMFS1* enables better yielding traits than ectopic activation of *VRT-A2*. **A)** Glume and grain phenotypes of wild type (WT), *Tamfs1*, Fielder*^A2b^* (the *VRT-A2b*-introgression line at the genetic background of Fielder), and *VRT-A2*-OE (*VRT-A2* overexpression transgenic plants) collected in field growth condition. Scale bars, 1 cm. **B)** Statistical analyses of the agronomic and yielding traits of WT, *Tamfs1,* Fielder*^A2b^*, and *VRT-A2*-OE as shown in **A)**. Data are means ± Sd. Plants used for phenotypic analyses and data collection were sampled in the plots planted in a field at the China Agricultural University Experimental Station (Beijing, People's Republic of China) in the 2023 to 2024 growing season. For the determination of plant height, spike length, glume length, grain length, grain width, TGW, and grain yield per spike, *n* = 50 main spikes randomly collected from 5 biologically independent plots; for TGW, grain length, and grain width, *n* = 50 individual plants randomly collected from 5 biologically independent plots, and the grains of the plants were all harvested for phenotypic analyses. **C)** RT-qPCR analysis showing the relative expression levels of *VRT-A2* in WT, *Tamfs1*, Fielder*^A2b^*, and *VRT-A2*-OE lines. The spikes of WT, *Tamfs1*, Fielder*^A2b^*, and *VRT-A2*-OE were collected from the plants grown in a growth chamber under long-day photoperiods (16-h-light/8-h-dark, 25 °C/18 °C) at different developmental stages. Data are means ± Sd (*n* = 3 biologically independent replications). Different lowercase letters in **B)** indicate significant differences at *P* < 0.05 (1-way ANOVA with Tukey's multiple comparisons test). Asterisks in **C)** represent significant differences between WT and the materials including *Tamfs1,* Fielder*^A2b^*, and *VRT-A2*-OE based on 1-way ANOVA followed by Dunnett's tests (****P < 0.0001; ***P < 0.001; **P < 0.01; *P < 0.05).

Since TaMFS1 and TaSSRP1 play opposite roles in regulating *VRT-A2* expression, we deleted the in vivo *TaMFS1* gene through CRISPR/Cas9 gene editing system, aiming to tip the TaMFS1–TaSSRP1 balance toward TaSSRP1. Totally, we obtained 2 independent *Tamfs1* null mutant lines (#1 and #2) under the genetic background of Fielder, in which all 3 homoeologs of *TaMFS1* (from A, B, and D subgenomes) were simultaneously knocked out (Supplementary Figs. S16 and S17), and tested their agronomic traits in field growth condition. As expected, all these mutant lines exhibited significantly longer glumes and grains than that of WT but showed similar grain width as in WT (Fig. 6, A and B). In support of these observations, RT-qPCR assays revealed that the expression levels of *VRT-A2* were higher in the spike tissues of *Tamfs1* mutant lines than that in WT (Fig. 6C), again supporting the in vivo repression effect of TaMFS1 on *VRT-A2* transcription. We also detected significant upregulation of the transcript levels of *VRT-B2* and *VRT-D2*, in the spike tissues of *Tamfs1* mutant lines when compared to that in WT spikes (Supplementary Fig. S18). These findings suggest a highly conservative biological effect of TaMFS1 in regulating the *VRT2* homoeologous genes.

**Figure 7.**
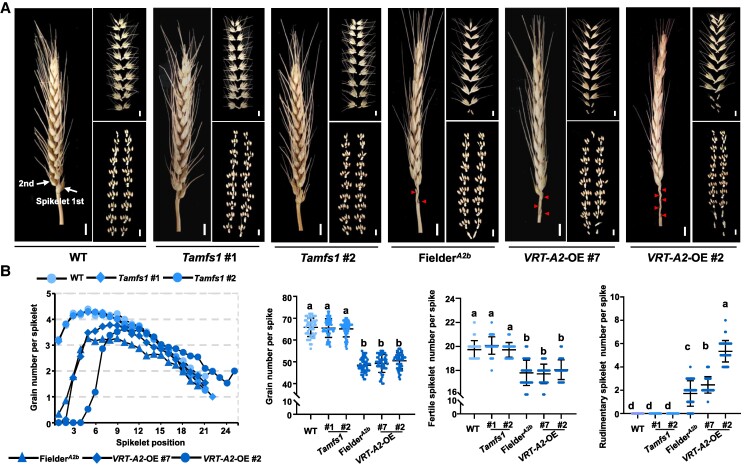
Fielder*^A2b^* and *VRT-A2-*OE, but not *Tamfs1*, show increased rudimentary spikelet number and decreased GNS. **A)** The spikes of WT, *Tamfs1* mutants, Fielder*^A2b^*, and *VRT-A2* overexpression (*VRT-A2*-OE) lines at mature stage. Scale bars, 1 cm. Triangles indicate the rudimentary basal spikelets. Images were digitally extracted for comparison. **B)** Statistical analyses of the spike traits, including grain number per spikelet, GNS, fertile spikelet number per spike, and rudimentary spikelet number per spike. Plants for phenotypic analyses and data collection were sampled in the plots planted in a field at the China Agricultural University Experimental Station (Beijing, People's Republic of China) in the 2023 to 2024 growing season. For statistical analyses, *n* = 50 main spikes from 50 independent plants. For the determination of grain number per spikelet, the grains were numbered from bottom to top, with the 1st spikelet at the bottom of the spike identified as 1. Different lowercase letters in **B)** indicate significant differences at *P* < 0.05 (1-way ANOVA with Tukey's multiple comparisons test). Data are means ± Sd.

By comparing the agronomic and yielding traits of *Tamfs1*, Fielder*^A2b^* (the *VRT-A2b*-introgression line at the genetic background of Fielder; see the “Materials and methods” section) and the previously generated *VRT-A2*-OE transgenic lines (Liu et al. 2021), we found that all these materials exhibited significantly increased grain length and TGW when compared with WT (Fig. 6, A and B). However, Fielder*^A2b^* and *VRT-A2-*OE also displayed undesirable traits, such as increased rudimentary basal spikelet number per spike and decreased GNS, which were not observed in *Tamfs1* (Fig. 7). As a result, based on the yielding traits collected from individual plants, *Tamfs1* harvested significantly higher grain yield per spike than Fielder*^A2b^* or *VRT-A2*-OE (Fig. 6B). Accordingly, we conclude that deleting *TaMFS1* may define a better strategy than direct overexpression of *VRT-A2* in improving yield performance of wheat.

## Discussion

In the previous study, we showed that TaMFS1 strongly represses the expression of *VRT-A2*, especially the WT *VRT-A2a* allele, depending on its binding to the intact GCC boxes enriched in the Intron-1 region (Liu et al. 2021). However, this repression effect of TaMFS1 was not well supported by the genetic evidence, due to the lack of *TaMFS1*-related transgenic wheat plants. In this study, we generated *TaMFS1*-OE transgenic lines and *Tamfs1* null mutant lines, aiming to comprehensively evaluate the in vivo biological significance of *TaMFS1*. As expected, in *TaMFS1*-OE plants, the expression of *VRT-A2* was strongly repressed (Fig. 1C; Supplementary Fig. S1B), while in *Tamfs1* mutants, higher *VRT-A2* expression was detected when compared with that in WT (Fig. 6C). These results support a negative correlation between TaMFS1 activity and *VRT-A2* expression. Moreover, ChIP-PCR assay revealed that the TaMFS1 proteins were enriched on the Intron-1 region of *VRT-A2* (Fig. 1D), confirming a direct binding of TaMFS1 with the gene body of *VRT-A2* in vivo.

It is well known that some TFs, especially those harboring the EAR motifs, always recruit the transcriptional corepressor proteins TPL, which function in combination with the histone deacetylase HDACs, to generate condensed chromatin and consequently inhibiting target gene expression (Hollender and Liu 2008; Shen et al. 2015; Plant et al. 2021). Indeed, our data revealed that TaMFS1 could directly interact with the TPL and HDAC homologs in wheat, such as TaTPL and TaHD2A (Fig. 2). Consistent with these findings, we revealed significantly altered epigenetic modification patterns at the *VRT-A2* gene locus in *TaMFS1*-OE plants relative to that in WT (Fig. 2M; Supplementary Fig. S6). These findings together support the conservativeness of the TPL–HDAC module in modifying local chromatin states and thus facilitating the transcriptional repression activities of the EAR motif-containing TFs in different plant species.

The timely suppression of the transcription of *VRT2* and its closely related *SVP* genes represents the strictly controlled vegetative-to-reproductive phase transition in multiple plant species (Brill and Watson 2004; Masiero et al. 2004; Sentoku et al. 2005; Trevaskis et al. 2007; Li et al. 2019; Li et al. 2021). This has led to a concern that direct engineering of the phase transition gene *VRT-A2*, either through the overexpression or through the knockout of the functional copies of this gene, may easily cause plant abnormalities, especially during the reproductive process. Indeed, in Polish wheat, strong and ectopic activation of *VRT-A2* leads to increased number of rudimentary basal spikelets and decreased grain number per plant (Watanabe et al. 1996; Okamoto and Takumi 2013), resulting in yield penalties. This nature of *VRT-A2* has hindered its direct use in wheat breeding practice. Similar situations have also been noticed in other important genes, for example *IDEAL PLANT ARCHITECTURE 1* (*IPA1*) in rice and the *CLAVATA-WUSCHEL* module in tomato (*Solanum lycopersicum*) (Jiao et al. 2010; Miura et al. 2010; Rodriguez-Leal et al. 2017). Previous studies have confirmed that optimizing the expression levels or tissue specific patterns of these pleiotropic genes, either by editing the promoter sequences or by modifying the regulatory factors, may serve as feasible strategies to overcome their tradeoffs and thus improving crop yield performance without penalties (Wang et al. 2017; Zhang et al. 2017; Song et al. 2022). Here in our study, by editing *TaMFS1*, the upstream regulator of *VRT-A2*, we successfully elevated *VRT-A2* expression in a moderate manner in these *Tamfs1* mutant lines (Fig. 6C). Importantly, these *Tamfs1* lines show significantly increased grain length and enhanced grain weight than in Fielder, largely phenocopying that in *VRT-A2*-OE plants; whereas unlike the overexpression of *VRT-A2* that triggers the increase of rudimentary basal spikelets, *TaMFS1* knockout shows no detectable pleiotropic effect on spikelet development (Fig. 6, A and B). As a result, *Tamfs1* lines show significantly higher grain yield per spike than Fielder (Fig. 6B). This indicates that, through engineering *TaMFS1*, we can moderately activate the expression of *VRT-A2* to improve the spike grain yield of wheat, thus providing a feasible strategy and an indirect way to use *VRT-A2* for the molecular design of high-yield wheat cultivars.

It has long been known that the introns, especially the long 1st intronic DNA sequences, tend to harbor regulatory elements and can strongly facilitate gene expression, and this phenomenon is termed IME (Gallegos and Rose 2015; Shaul 2017; Zalabák and Ikeda 2020). Several conserved motifs responsible for IME have been defined in previous studies in Arabidopsis and rice (Rose et al. 2008; Morello et al. 2011). However, until present, few protein factors have been functionally characterized to potentially associate with these IME motifs and contribute to the IME-related gene activation. *VRT-A2* has a very long first intron, among which we indeed identified a typical IME sequence (GATCTGT; 1 copy in the WT *VRT-A2a* allele, while 6 copies in the Polish wheat–derived autoactivated *VRT-A2b* allele), which shows homologous to the IME elements defined in Arabidopsis (Rose et al. 2008; Liu et al. 2021). In this study, we show that the structure-specific recognition protein TaSSRP1 could directly associate with the IME motifs identified in the *VRT-A2* Intron-1 regions (Fig. 3). Importantly, TaSSRP1 activates *VRT-A2* expression in an IME motif-dependent manner (Fig. 3). These results strongly support that TaSSRP1 is a factor contributing to IME. Consistent with its protein identity in facilitating gene expression, TaSSRP1 recruits Mediator and RNA Pol II (Fig. 5; Supplementary Fig. S14), which are thought to be crucial for the transcriptional initiation and elongation, and thus influencing gene transcription efficiency (Allen and Taatjes 2015; Buendía-Monreal and Gillmor 2016). Notably, the assembly of RNA Pol II into the punctate structure of TaSSRP1 in the nucleus depends strictly on the presence of TaMED6 (Fig. 5; Supplementary Fig. S14), suggesting that in such TaSSRP1–TaMED6–RNA Pol II complex, TaMED6 is a key factor bridging TaSSRP1–RNA Pol II association. Recent years, growing studies have highlighted the essential roles of the compartmentalization and concentration of functionally related transcriptional components in activating or repressing gene expression (Hnisz et al. 2017; Boija et al. 2018; Sabari et al. 2018; Huang et al. 2021, 2022; Wang et al. 2023). The discovery of the “TaSSRP1–TaMED6–RNA Pol II” module in this study not only deepens our current understanding of the IME regulatory mechanism in plant species, but also provides a framework for better assessing the impact of concentration of transcriptional components in facilitating gene expression in crops.

In summary, our findings highlight a sophisticated transcriptional regulation of *VRT-A2* by 2 contrasting signaling modules, i.e. the “TaMFS1–TaTPL–TaHD2A” repression complex and the “TaSSRP1–TaMED6–RNA Pol II” activation complex (Supplementary Fig. S19). In addition, our current efforts in editing *TaMFS1* has provided a selectable strategy to overcome the tradeoffs between spikelet repression and grain weight enhancement caused by the pleiotropic effects of *VRT-A2* activation, we believe that the potential of *VRT-A2* in enhancing grain weight has not been fully explored. Better understanding of TaMFS1–TaSSRP1 balance may help us to further optimize *VRT-A2* expression in wheat spike and grain tissues, thus enables a better utilization of this gene in wheat high-yield breeding practice by eliminating its shortcomings.

## Materials and methods

### Plant materials and growth conditions

To evaluate the phenotypic effects of *VRT-A2b* in spring wheat c.v. Fielder background, the hexaploid *T. petropavlovskyi* accession 3989 was backcrossed with Fielder (serving as the recurrent parent) for 5 rounds to obtain the *VRT-A2b*-introgression line Fielder*^A2b^* (harboring the homozygous *VRT-A2b* allele). Fielder was also used as the background for gene transformation and gene editing.

For pot experiments, the wheat seeds of transgenic and gene-edited T_0_, T_1_, and T_2_ generation plants and the WT Fielder were germinated and placed at room temperature for 3 d and then were transferred into the soil-filled plastic pots (1 seed for 1 pot). Each material had 3 replicates. For each replicate, 5 or 11 plants were selected for agronomic trait evaluation. The pot-grown seedlings were transferred into a growth chamber (400 W HPS lamps, light intensity of 3,000 lux) with 16-h-light/8-h-dark photoperiod (25 °C/18 °C, 65% relative humidity) at China Agricultural University. All pots were randomly placed according to the completely randomized design.

Field experiments were performed in randomized plot design with 5 replications. In each replication, wheat seeds were hand planted in 5-row plots of 1.5 m in length spaced 0.30 m apart with 20 seeds per row. In each experiment, 5 plots were planted independently for one material, which were considered as 5 replications. The field experiments were conducted in a field at the China Agricultural University Experimental Station (Beijing, People's Republic of China) in the 2023 to 2024 growing season. The plants were grown in a standard field growth condition, following local agricultural practices for field management and avoiding floods, droughts, pests, or diseases. Wheat materials in the middle part of the rows were randomly selected for phenotypic analyses.

*N. benthamiana* plants were planted in the greenhouse under a 16-h light and 8-h dark photoperiod at 22 °C.

### Gene transformation in wheat

The full-length coding sequences (CDSs) of *VRT-A2*, *TaMFS1*, and *TaSSRP1* genes were amplified from the complementary DNA (cDNA) of Fielder, and the PCR fragments together with the 3× Flag or GFP tag sequences were cloned into the *BamHI*-digested plant binary vector *pWMB110* to fuse with the *Ubiquitin* promoter from maize (*Z. mays*). The generated vectors were introduced into *Agrobacterium tumefaciens* strain EHA105 and then were transformed into Fielder (Kumar et al. 2019). In T_0_ generation, the DNA insertion was validated by PCR; in T_1_ and T_2_ generations, total RNAs were extracted from the leaves of the transgenic plants when the spikes were about 0.5 or 3.5 cm in length, and the expression levels of the transformed genes were determined.

Alternatively, the total proteins were extracted from the leaves of wheat seedlings for subsequent Western blotting assays using the anti-Flag (1:5,000; Sigma, F1804) antibody. Primers used for gene cloning and plasmid construction are listed in Supplementary Data Set 3, and the ID numbers of genes are listed in Supplementary Data Set 4.

### CRISPR/Cas9-mediated gene editing

For generating *Tassrp1* and *Tamfs1* mutant plants by CRISPR/Cas9, about 23-bp sgRNA target sequences (including Protospacer Adjacent Motif) were designed according to the exon sequences of *TaSSRP1* and *TaMFS1* based on the online software E-CRISPR (http://www.e-crisp.org/E-CRISP/). The specificity of these targeting sequences was evaluated by a Blast search against the wheat genome (http://plants.ensembl.org/Triticum_aestivum/Tools/Blast). Then, the target sequence fragments were separately cloned into the binary vector *pBUE414*, which were further introduced into *A. tumefaciens* (strain EHA105) for gene transformation. The genomic DNA samples were extracted from the transgenic lines, and the primers flanking the designated target sites were used for the confirmation of the mutations. Primers used for the detection of gene mutations are listed in Supplementary Data Set 3.

### Phenotypic analysis

The agronomic traits including plant height, glume length, spike length, total spikelet number per spike, fertile spikelet number per spike, and rudimentary spikelet number per spike were measured manually before harvest. The grain traits including TGW, grain length, grain width, and grain yield per spike were analyzed after harvest.

For glume length measurement in pot experiments, 5 or 11 main spikes were selected in each replicate of each line, and totally 15 or 33 main spikes from 3 replicates were collected for statistical analyses. In each spike, we collected 6 glumes from the central 3 spikelets (9th to 11th spikelets from the bottom) of the main spike and measured their lengths with a vernier caliper. The lengths of these 6 glumes were averaged for further analysis. To evaluate the grain traits including grain length and grain width in pot experiments, 5 or 11 independent wheat plants were randomly selected in each replication, and totally 15 or 33 independent plants from 3 replications were manually harvested, and these harvested grains were all sampled for statistical analyses with a Wanshen SC-G seed detector (Hangzhou Wanshen Detection Technology).

For the measurement of plant height, spike length, glume length, total spikelet number per spike, fertile spikelet number per spike and the number of rudimentary spikelet per spike in field plot experiments, 10 main spikes/culms were randomly selected in each plot, and totally 50 main spikes/culms from 5 independent plots were collected for statistical analyses. To evaluate the grain traits in field plot experiments, 10 independent wheat plants were randomly selected in each plot of each line, and totally 50 independent plants were manually harvested. All harvested grains were used for statistical analyses with a Wanshen SC-G seed detector (Hangzhou Wanshen Detection Technology). To determine the grain yield per spike in field plot experiments, 10 main spikes were randomly sampled in each plot of each material, and totally 50 main spikes were collected from 5 independent plots. Then, the grains from each main spike were harvested manually and weighted with an electronic balance.

### RNA extraction and RT-qPCR analysis

Plants used for RNA extraction were grown in a growth chamber under long-day photoperiods (16-h-light/8-h-dark, 25 °C/18 °C). To determine the spatial expression patterns of *TaSSRP1*, *TaHD2A*, *TaTPL*, and *TaMED6* genes, different tissues of Fielder including roots, stems, leaves, and spikes were collected for analyses. To detect the expression levels of *TaSSRP1* and *VRT-A2* in *TaSSRP1*-OE transgenic plants, 3.5-cm spike of WT and *TaSSRP1*-OE were collected. To detect the expression levels of *VRT-A2* and its homoeologs (*VRT-B2* and *VRT-D2*) in WT, *VRT-A2-*OE, Fielder*^A2b^*, *Tamfs1*, and *TaMFS1-*OE plants, leaf samples or spikes at different developmental stages (0.5, 1, 1.5, 2, 2.5, 3.0, 3.5, 4.0, and 4.5 cm in length) were collected for RNA extraction. For each experiment, 3 independent biological replications were carried out with similar results.

Total RNA was extracted using Trizol reagent (Invitrogen) according to the manufacturer's protocol. The genomic DNA (gDNA) was removed with genomic DNA Wiper, and the 1st-strand cDNA was synthesized using 1 *µ*g total RNA and a reverse transcription kit (Vazyme Biotech, R223). Quantitative real-time PCR (RT-qPCR) was carried out in a 10-*μ*L volume containing 1 *μ*L 5-fold diluted cDNA template and 5 *μ*L SYBR Green PCR Master Mix (Q121, Vazyme Biotech) with a Real-Time System (CFX96, Bio-Rad). The *Actin* gene (*TraesCS5B02G124100*) was used as an internal control.

### ChIP assay

The leaf samples of WT and *TaMFS1-*OE were collected when the spikes were about 0.5 cm in length, and the 3 to 4 cm spikes of WT and *TaSSRP1*-OE were collected for ChIP assay. The ChIP assay was conducted as described previously (Liu et al. 2021). In brief, ∼2 to 3 g of samples were ground into powder in liquid nitrogen and cross-linked with 1% (v/v) formaldehyde for 10 min, and then the reaction was stopped by 0.1 m glycine for 5 min. After nucleus lysis and chromatin isolation, the genomic DNA was sonicated to produce fragments with a size of ∼500 bp. The supernatants were preincubated with protein A/G agarose beads (Sigma-Aldrich,16-663) for 1 h. Immunoprecipitation was carried out with the Flag M2 (Sigma, F1804), anti-GFP (Abcam, ab290), anti-H3K9ac (catalog no. 07-352, Millipore), or anti-H3K27me3 (catalog no. 17-622, Millipore) antibodies at 4 °C overnight. The precipitated samples without antibody were used as negative controls, while the samples before precipitation were used as the input controls. ΔCt was calculated by comparing the threshold cycle number (Ct) for the ChIP DNA fraction and the input sample to represent relative enrichment percentage (Asp 2018). The values of negative control samples were set to 1. The primers used for ChIP-PCR are listed in Supplementary Data Set 3.

### Subcellular localization

To investigate the subcellular localization of TaMFS1, TaTPL, TaHD2A, TaSSRP1, TaMED4, TaMED6, TaMED8, TaMED25, and TaCTD, their CDSs without stop codons were separately cloned into the *pCAMBIA super1300-GFP*, *pCAMBIA super1300-BFP*, or *pCAMBIA super1300-mCherry* vectors and then were transferred to the p19-containing *A. tumefaciens* strain GV3101 (pSoup-p19) for further infiltration in *N. benthamiana* leaf samples. Fluorescence was examined under a confocal laser-scanning microscope (LSM880, Zeiss, Germany) at 36 h post infiltration with a laser intensity of 2% to 10%, and images were processed and analyzed by ZEN Microscopy Software (Zeiss). BFP, GFP, and mCherry were excited at 405, 488, and 543 nm, respectively, and detected at 410 to 530, 490 to 579, and 579 to 650 nm, respectively. The master gain value was no more than 800, digital gain value was set to 1, and the pinhole was set to 1 AU. The primers used in this assay are listed in Supplementary Data Set 3.

### Phylogenetic analysis

The homologous proteins were searched in Ensembl Plants database (http://plants.ensembl.org/) and NCBI (https://www.ncbi.nlm.nih.gov/gene/). Sequence comparison and phylogenetic tree construction analysis were conducted via MEGA 7.0 with bootstrap values of 1,000 replicates. The sequences for alignment are provided as Supplementary Files 1 and 2.

### Y2H assay

Yeast 2-hybrid assays were performed as described in the Yeast Protocols Handbook (Clontech). The full-length CDS of *TaMFS1* was cloned into the *pGBKT7* (BD) vector, and the recombinant plasmid and *pGADT7* (AD) EV were cotransformed into the Y2HGold yeast strain to test for self-activation. Subsequently, *TaMFS1* was used as the bait protein to screen against a Y2H cDNA library generated from young wheat spike tissues, and candidate clones were obtained through sequencing and alignments. The CDS of *TaTPL* was introduced into the vector *pGADT7* to generate AD-TaTPL, while the full-length CDS of *TaMFS1* was inserted into the *pGBKT7* vector to generate BD-TaMFS1. AD-TaTPL and BD-TaMFS1 were coexpressed in the Y2HGold strain cells and selected on the synthetic dextrose (SD) media lacking Leu, Trp, His, and Ade (SD-L/W/H/A) (Clontech). The empty AD and BD were served as negative controls. The primers used in this assay are listed in Supplementary Data Set 3.

### Co-IP assay

The full-length CDSs of *TaMFS1* and *TaTPL* were amplified by PCR from Chinese Spring cDNA and cloned into *pCAMBIA1300-GFP* and *pCAMBIA1300-Myc* vectors to separately produce the *TaMFS1- GFP* and *TaTPL-Myc* vectors. The above vectors were transformed into the *A. tumefaciens* strain GV3101. Next, the *A. tumefaciens* strains harboring different expression vectors were coinfiltrated with indicated combinations into the *N. benthamiana* leaves. Leaf samples were collected 48 h postinfiltration for Co-IP assay. Total proteins were extracted using lysis buffer containing 50 mm Tris-HCl at pH 7.5, 150 mm NaCl, 5 mm EDTA at pH 8.0, 0.1% Triton X-100, 0.2% NP-40, 0.6 mm phenylmethylsulfonyl fluoride (PMSF), and protease inhibitor cocktail. The samples were centrifuged at 12,000 × *g* at 4 °C for 30 min. Anti-GFP-conjugated magnetic agarose beads (gtma-20, Chromotek) were used for immunoprecipitation. The immunoprecipitated samples were washed with washing buffer (50 mm Tris-HCl, pH 7.5, 150 mm NaCl, and 0.1% NP-40) for 5 times and then were separated on SDS-PAGE gel and subjected to immunoblot analysis with anti-Myc (mouse; 1:2,000 dilution; California Bioscience, CB100002M) and anti-GFP (rabbit; 1:2,000 dilution; Abcam, ab32146) antibodies.

### LCI assay

The full-length CDSs of *TaMFS1*, *TaTPL*, *TaHD2A*, *TaHDA1*, *TaHDA6, TaSSRP1*, *TaMED6, TaMED25*, and *TaCTD* were separately cloned into the *pCAMBIA-1300-cLUC* and *pCAMBIA-1300-nLUC* binary vectors to generate the N-terminal LUC (nLUC) or C-terminal LUC (cLUC) fused proteins, respectively. The above expression vectors were transformed into the *A. tumefaciens* strain GV3101 and then were infiltrated into the *N. benthamiana* leaves for transient gene expression. After 36 to 48 h post infiltration, the LUC substrate D-luciferin (Promega, Cat # P1043) was sprayed onto the leaf surface, and the LUC activity was evaluated using the NightSHADE LB985 plant imaging system (Berthold Technologies, Germany). The primers used in LCI assays are listed in Supplementary Data Set 3. In each assay, 9 biologically independent *N. benthamiana* leaves collected from 9 independent plants were selected for statistical analysis.

### BiFC assay

For BiFC assay, the target genes were separately fused with the N-terminal or C-terminal yellow fluorescent protein (YFP)-CDSs to produce *cYFP-TaHD2A*, *nYFP-TaTPL*, *cYFP-TaSSRP1*, and *nYFP-TaMFS1* expression vectors. These constructs were further transformed into *A. tumefaciens* strain GV3101 for transient expression in *N. benthamiana* leaves. About 48 h postexpression, the YFP fluorescence was observed using a confocal laser-scanning microscope (LSM880, Zeiss). To capture fluorescence signals, the 488-nm excitation laser wavelength was used, and images were analyzed by ZEN Microscopy Software (Zeiss). Three biological replicates were conducted for each experiment. The primers used in BiFC assays are listed in Supplementary Data Set 3.

### In vitro pull-down assay

The CDSs of *TaMFS1*, *TaTPL*, *TaHD2A*, *TaSSRP1*, and *TaMED6* were cloned into the *pGEX-6P-1*, *pMAL-c2x* or *pET-32a* vectors to express MBP-TaMFS1, GST-TaMFS1, His-TaHD2A, MBP-TaTPL, His-TaSSRP1, and MBP-TaMED6 proteins in *E. coli* BL21(DE3) cells, respectively. The expressed proteins were purified using Ni Sepharose 6 Fast Flow resin (Cytiva, 17531801) or high-affinity amylose resin (NEB, E8021L). Biotin-H3 (1 to 21) and Biotin-H3K27me3 peptides used in this study were biochemically synthesized by Bankpeptide Bio-Tech Co., Ltd (Hefei, China) with a purity >95%. Biotin H3K9ac (cat. no: 81044) was obtained from Active motif.

The purified recombinant proteins were added to the pull-down incubation buffer (50 mm Tris-HCl, 100 mm NaCl, 0.6% Triton X-100, 0.2% glycerol, and 1 mm PMSF) with indicated combinations. The mixture was incubated with high-affinity amylose resin (NEB, E8021L) for 3 h at 4 °C with gentle shaking, and then the amylose resin was washed with the pull-down incubation buffer for 5 times. The immunoprecipitated proteins were eluted with 2× SDS extraction buffer (125 mm Tris-HCl, pH 6.8, 2% β-mercaptoethanol, 4% SDS, 20% glycerol, and 0.25% bromophenol blue) and then were detected by immunoblotting with anti-MBP (TransGen Biotech, HT701-01), anti-GST (TransGen Biotech, HT601), and anti-His (TransGen Biotech, HT501-01) antibodies. The peptide pull-down assays were performed as described previously (Wu et al. 2022).

### Transcriptional activation activity assay

The transcriptional activation activity assays were performed in *N. benthamiana* leaves as previously described (Liu et al. 2017). The DNA sequences were inserted into *pGWB35* vector through the Gateway reaction (Invitrogen) (Nakagawa et al. 2007), to fuse with the *LUC* reporter gene. The obtained vector was coexpressed with indicated protein factors in *N. benthamiana* leaf cells, and the luminescence images were captured by the Night SHADE LB985 (Berthold) with Indigo software. In each experiment, 9 independent *N. benthamiana* leaves were analyzed. The primers used in this assay are listed in Supplementary Data Set 3.

### Electrophoretic mobility shift assay

The full-length CDSs of TaSSRP1 were amplified and separately cloned into the vector *pET-32a* and transformed into *E. coli strain* Transetta (DE3). The recombinant proteins were purified by usingNi Sepharose 6 Fast Flow resin (Cytiva, 17531801). DNA probes were artificially synthesized and 5′-end were labeled with biotin. Labeled probes of 1 *μ*L (1 ng) were incubated with 2 *μ*g of purified recombinant proteins at room temperature (24 to 28 °C) for 20 min in a binding reaction mixture of 20 *μ*L with 1× binding buffer, 2.5% glycerol, 2 mm EDTA, 5 mm MgCl_2_, 50 mm KCl, 0.05% NP-40, and 50 n poly (dI-dC). For competition assays, 10-, 50-, and 100-fold unlabeled probes were added to the reaction mixture. DNA gel shift assays were conducted according to the manual for the LightShift Chemiluminescent EMSA Kit (Thermo Fisher Scientific, 20148). The primers used in EMSA assays are listed in Supplementary Data Set 3.

### Statistical analysis

Statistical analyses of all bar graphs were performed using GraphPad Prism (Version 8.0.2) or Microsoft Excel (2010). Values were represented as mean ± Sd. Significant differences between 2 groups were tested with 2-sided Student's paired *t-*test by using Microsoft Excel 2010 software. Differences between 3 or more groups were analyzed by 1-way ANOVA followed by Tukey's multiple comparison test or Dunnett's multiple comparison test with GraphPad Prism 8 software. Details of statistical analyses including analytical methods, *n* values, and significance levels (*P-*values) can be found in the figures, figure legends, or Supplementary Data Set 5.

### Accession numbers

All genes’ information in this article can be found in the GenBank/EMBL data libraries under accession numbers described in Supplementary Data Set 4.

## Supplementary Material

koaf024_Supplementary_Data

## Data Availability

The data underlying this article are available in the article and in its online Supplementary material.

## Supplementary data

The following materials are available in the online version of this article.

**Supplementary Figure S1.** The accumulation of TaMFS1 and the expression of VRT-A2 and SVP-A1 in TaMFS1-OE transgenic wheat plants (supports Fig. 1).

**Supplementary Figure S2.** Phylogenetic analysis of the protein sequences of HDACs from wheat and Arabidopsis (supports Fig. 2).

**Supplementary Figure S3.** TaMFS1 does not interact with TaHDA1-A or TaHDA6-A (supports Fig. 2).

**Supplementary Figure S4.** Subcellular localization of TaTPL, TaHD2A, and TaMFS1 in *N. benthamiana* leaves (supports Fig. 2).

**Supplementary Figure S5.** Expression patterns of TaMFS1, TaTPL, and TaHD2A in different wheat tissues (supports Fig. 2).

**Supplementary Figure S6.** Local histone trimethylation modification of lysine 27 (H3K27me3) at the VRT-A2 locus in TaMFS1-OE lines (supports Fig. 2).

**Supplementary Figure S7.** Phylogenetic analysis of TaSSRP1 orthologs in different plant species and the expression pattern of TaSSRP1 in different wheat tissues (supports Fig. 3).

**Supplementary Figure S8.** Partial Intron-1 sequences of VRT-A2 used to design the EMSA probes (supports Fig. 3).

**Supplementary Figure S9.** TaSSRP1 does not influence grain width in wheat (supports Fig. 4).

**Supplementary Figure S10.** Generation of Tassrp1 mutant lines and their grain phenotype (supports Fig. 4).

**Supplementary Figure S11.** TaSSRP1 influences histone H3 acetylation (H3K9Ac) and H3 trimethylation of lysine 27 (H3K27me3) at the VRT-A2 locus (supports Fig. 4).

**Supplementary Figure S12.** Subcellular localization of the GFP- or BFP-tagged TaSSRP1, TaMED4, TaMED6, and TaMED8 proteins (supports Fig. 5).

**Supplementary Figure S13.** Subcellular colocalization of TaSSRP1 and the Mediator subunits TaMED25, TaMED4 and TaMED8 (supports Fig. 5).

**Supplementary Figure S14.** Subcellular colocalization of TaSSRP1, TaMED6, and the C terminus of wheat RNA Polymerase II (TaCTD) (supports Fig. 5).

**Supplementary Figure S15.** RT-qPCR analysis showing the expression pattern of TaMED6 in different tissues (supports Fig. 5).

**Supplementary Figure S16.** Alignment of the CDSs of TaMFS1 homoeologs derived from the A, B, and D subgenomes, respectively (supports Figs. 6 and 7).

**Supplementary Figure S17.** The CRISPR/Cas9-triggered mutations in TaMFS1-A, TaMFS1-B, and TaMFS1-D for the generation of Tamfs1 mutant lines (supports Figs. 6 and 7).

**Supplementary Figure S18.** RT-qPCR analysis reveals the expression patterns of VRT-B2 and VRT-D2 in spike tissues of different lengths (supports Figs. 6).

**Supplementary Figure S19.** A proposed working model.

**Supplementary Data Set 1.** The TaMFS1-interacting protein candidates identified by Y2H.

**Supplementary Data Set 2.** Sequences used for the transactivation activity analysis.

**Supplementary Data Set 3.** Primers and probe sequences used in this study.

**Supplementary Data Set 4.** Genes analyzed in this study.

**Supplementary Data Set 5.** Summary of statistical analyses.

## Dive Curated Terms

The following phenotypic, genotypic, and functional terms are of significance to the work described in this paper:

Trp1 Gramene: AT5G59430
Trp1 Araport: AT5G59430

UBQ10 Gramene: AT4G05320
UBQ10 Araport: AT4G05320

ADH1 Gramene: AT1G77120
ADH1 Araport: AT1G77120

PAT1 Gramene: AT3G56920
PAT1 Araport: AT3G56920

SUS3 Gramene: AT4G02280
SUS3 Araport: AT4G02280

Act1 Gramene: Zm00001d010159
Act1 Araport: Zm00001d010159

MEGA CHEBI: CHEBI:6617
