# Supplementary material for: Decoupling the pleiotropic effects of VRT-A2 during reproductive development enhances wheat grain length and weight
Source: Plant Cell. 2025 Feb 14;37(2):koaf024. doi: 10.1093/plcell/koaf024 (PMC11827615; doi:10.1093/plcell/koaf024)
Supplement: koaf024_Supplementary_Data [file koaf024_supplementary_data.zip › Supplementary Data.pdf]

Supplementary Data

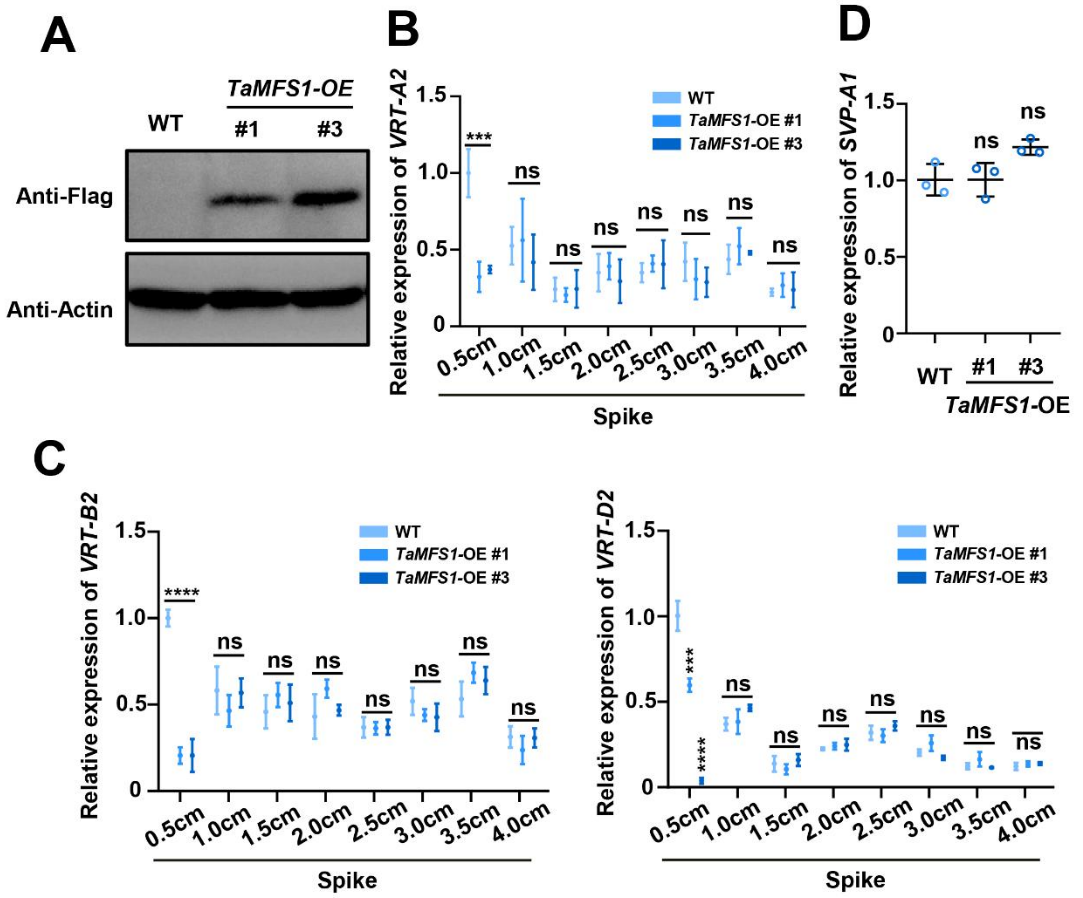

Supplementary Fig. S1 The accumulation of TaMFS1 and the expression of *VRT-A2* and *SVP-A1* in *TaMFS1*-OE transgenic wheat plants (Supports Figure 1).

A Western blotting showing the accumulation of TaMFS1-Flag proteins in the leaves of wild type (WT) Fielder and *TaMFS1* overexpression (*TaMFS1*-OE) lines (#1 and #3). Actin was used as an internal control for the assay. **B**, **C** The expression levels of *VRT-A2*, *VRT-B2* and *VRT-D2* in the spikes of WT and *TaMFS*1-OE transgenic lines. **D** The expression of *SVP-A1* gene in the leaves of WT and *TaMFS*1-OE transgenic lines. Data in **B**, **C** and **D** are means ± SD (n = 3 biologically independent replications). asterisks represent significant difference between WT and *TaMFS1*-OE based on one-way ANOVA followed by Dunnett's tests (**** $P < 0.0001$; *** $P < 0.001$). ns, no significant difference. The samples of WT and *TaMFS*1-OE were collected from the plants grown in a growth chamber under long-day photoperiods (16-h-light/8-h-dark, 25 °C/18 °C) at different developmental stages.

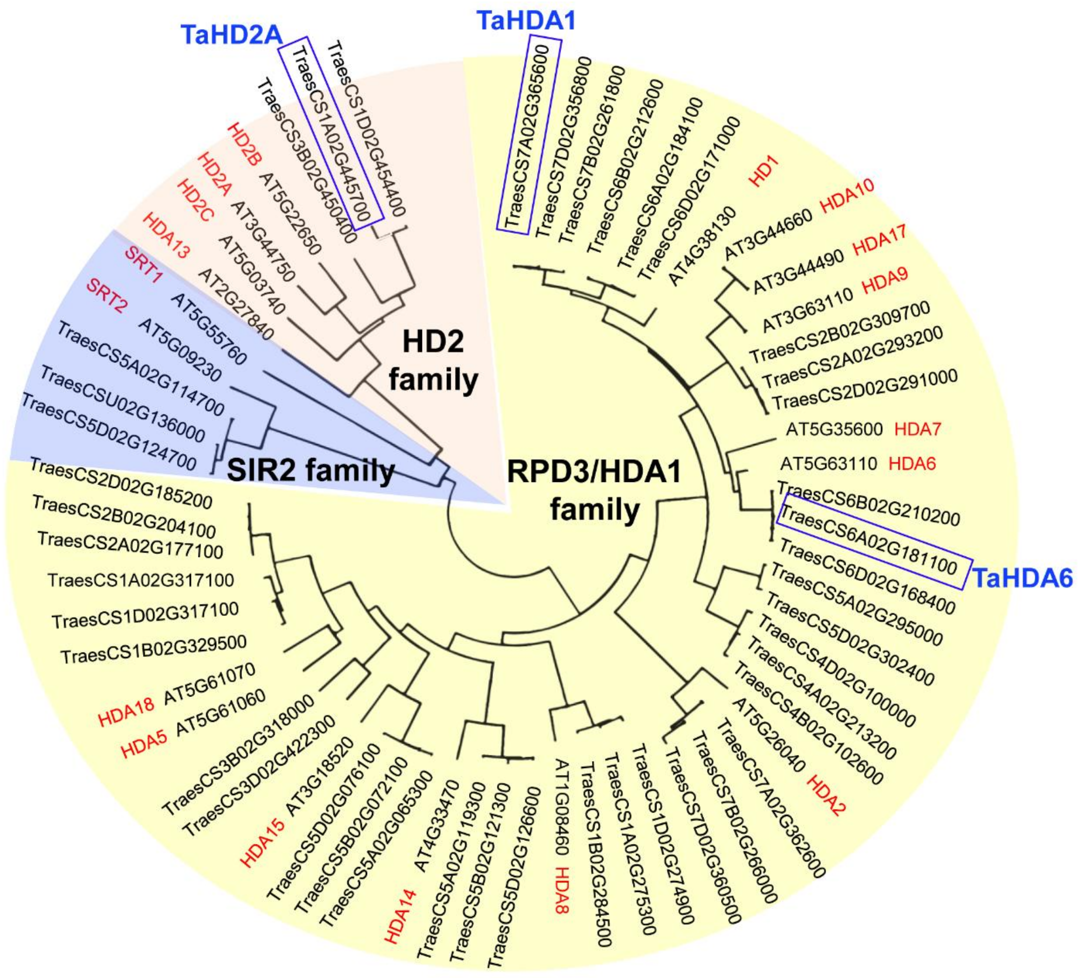

Supplementary Fig. S2 Phylogenetic analysis of the protein sequences of histone deacetylases (HDACs) from wheat and Arabidopsis (Supports Figure 2).
HDACs from Arabidopsis are shown in red, and three wheat orthologs of HDACs used in this study are highlighted in blue.

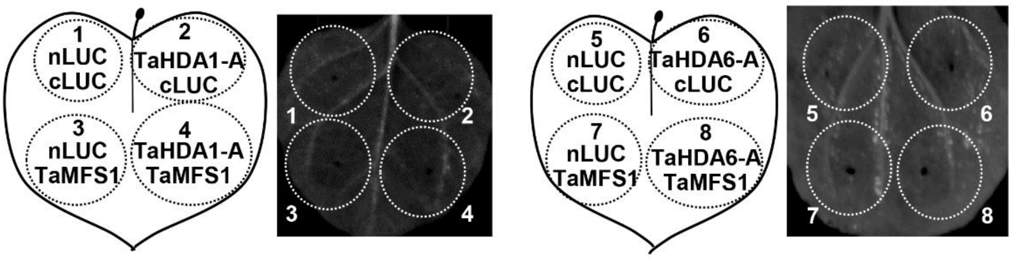

Supplementary Fig. S3 TaMFS1 does not interact with TaHDA1-A or TaHDA6-A (Supports Figure 2).

TaHDA1-A and TaHDA6-A were fused with the N-terminus of luciferase (nLUC), and TaMFS1 was fused with the C-terminus of LUC (cLUC). The nLUC and cLUC derivatives were co-expressed in *Nicotiana benthamiana* leaves with indicated combinations.

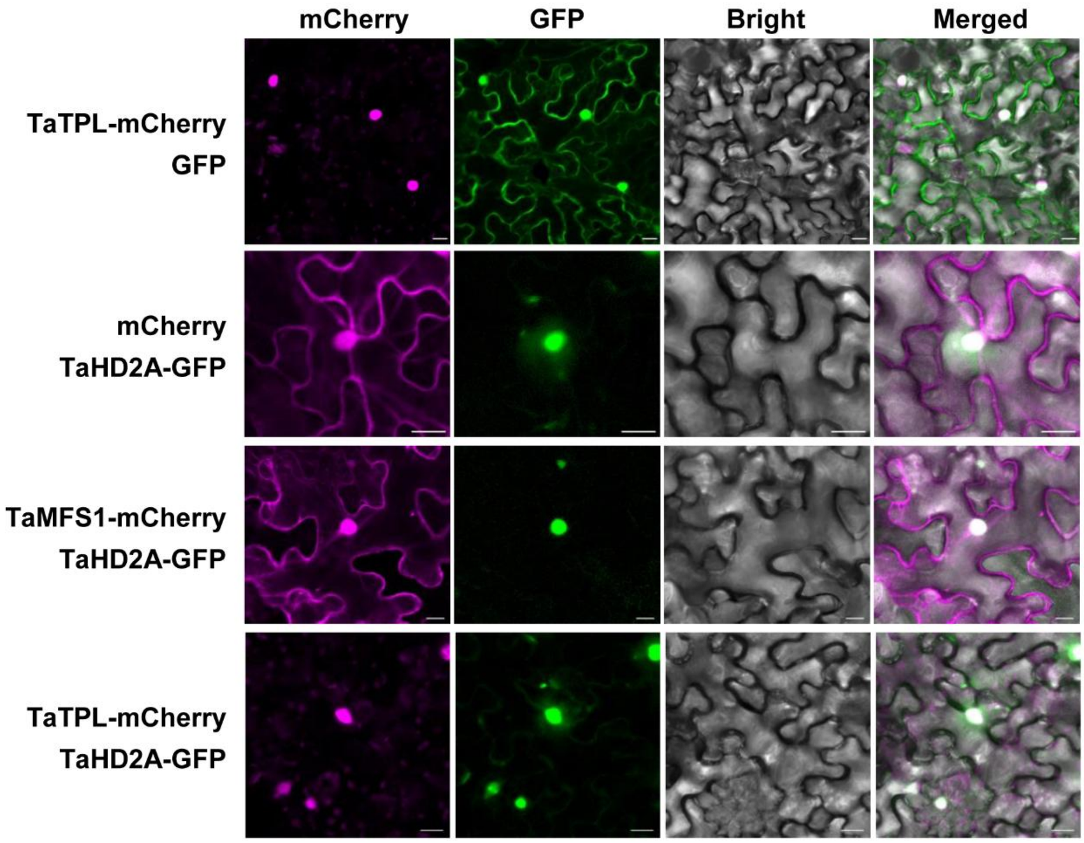

Supplementary Fig. S4 Subcellular localization of TaTPL, TaHD2A and TaMFS1 in *N. benthamiana* leaves (Supports Figure 2).

TaTPL-mCherry (magenta), TaHD2A-GFP (green) and TaMFS1-mCherry (magenta) were expressed in *N. benthamiana* leaves with indicated combinations, and the signals of mCherry and GFP were detected by confocal laser-scanning microscope. Bars = 20 μm.

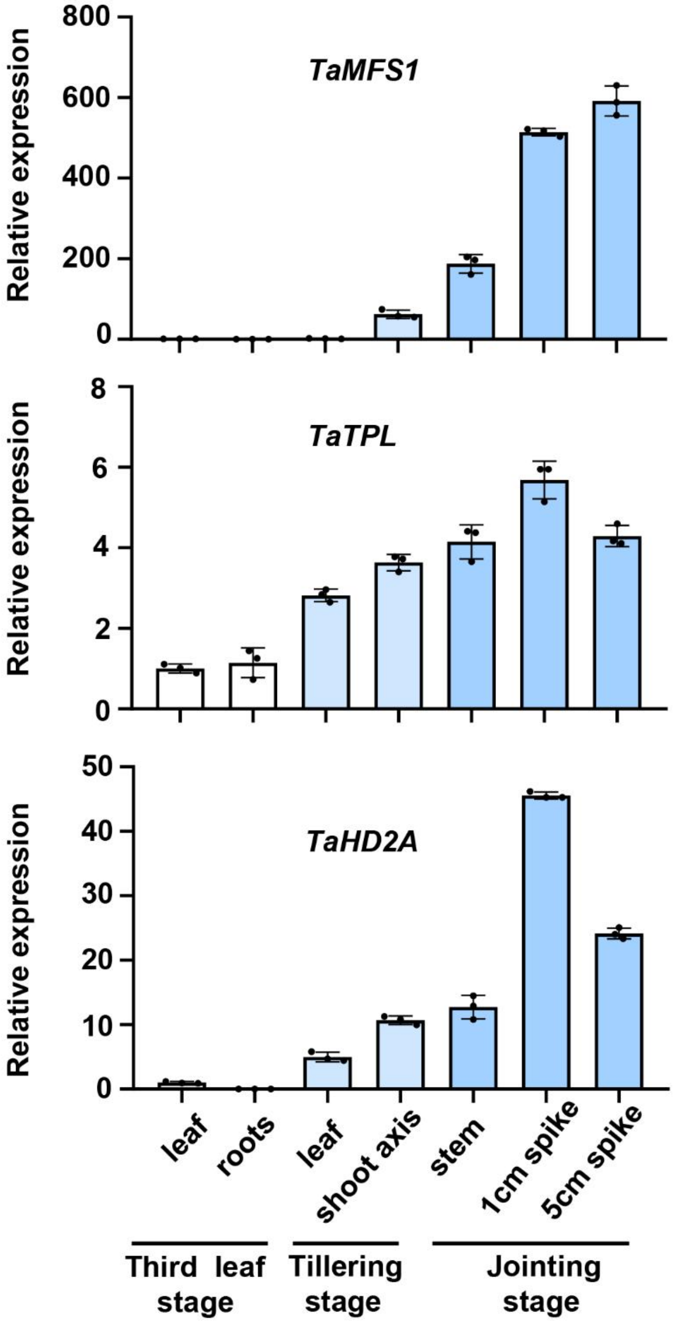

Supplementary Fig. S5 Expression patterns of *TaMFS1*, *TaTPL* and *TaHD2A* in different wheat tissues (Supports Figure 2).

Reverse transcription quantitative PCR (RT-qPCR) showing the expression levels of *TaMFS1*, *TaTPL* and *TaHD2A* in different wheat tissues, including roots, stems, leaves and spikes. Different tissues were collected from the pot-grown Fielder in a growth chamber. *Actin* was used as an internal control. Data are means ± SD (n = 3 biologically independent replications).

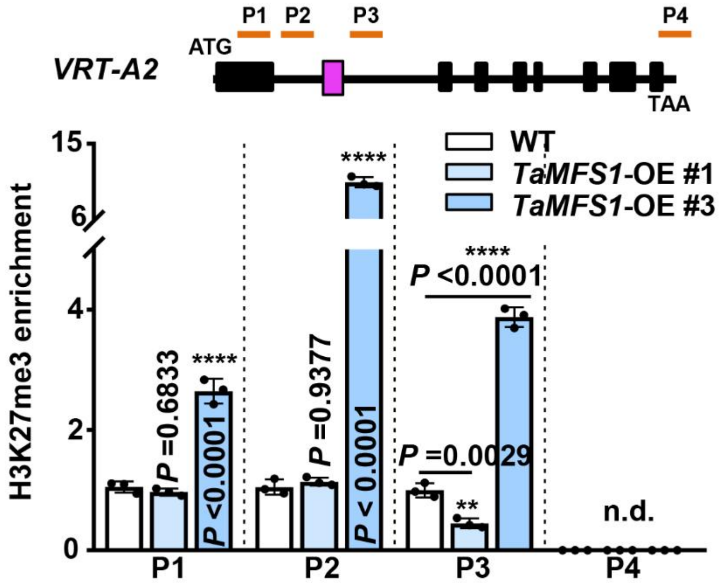

Supplementary Fig. S6 Local histone trimethylation modification of lysine 27 (H3K27me3) at the *VRT-A2* locus in *TaMFS1*-OE lines (Supports Figure 2). The leaf samples of wild type (WT) and *TaMFS1* overexpression (*TaMFS1*-OE) lines were collected for chromatin immunoprecipitation (ChIP) quantitative PCR (ChIP-qPCR) assay. A schematic representation of the gene structure of *VRT-A2* was shown above, and relative H3K27me3 enrichment levels on *VRT-A2* gene region were represented by the detection of four amplicons P1-P4. Data are means ± SD (n = 3 biologically independent replications). **** $P < 0.0001$; ** $P < 0.01$ (significant difference between WT and *TaMFS1*-OE based on one-way ANOVA followed by Dunnett's tests). n.d., not detected. The magenta box indicates the position of the 560-bp sequence specifically found in the intron-1 region of the *VRT-A2a* allele.

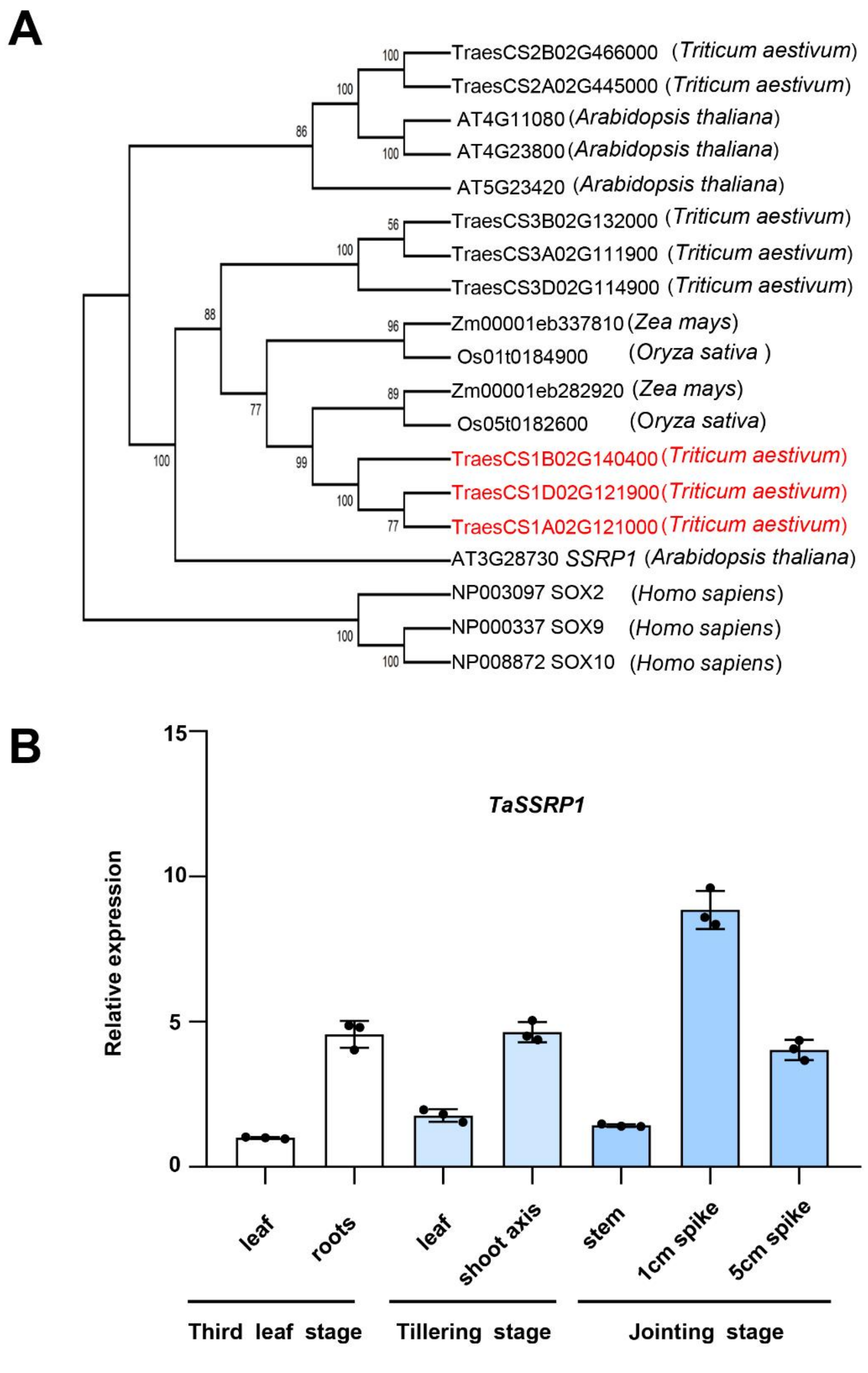

Supplementary Fig. S7 Phylogenetic analyses of TaSSRP1 orthologs in different plant species and the expression pattern of *TaSSRP1* in different wheat tissues (Supports Figure 3).

A Phylogenetic analysis of TaSSRP1 with its homologs from human, rice, maize, Arabidopsis and wheat. TaSSRP1 proteins from wheat are highlighted in red. Bootstrap support values (from 1,000 bootstraps) are shown at each node. **B** Relative expression levels of *TaSSRP1* in different tissues of wheat. Different tissues were collected from the pot-grown Fielder in a growth chamber. Data are means ± SD (n = 3 biologically independent replications).

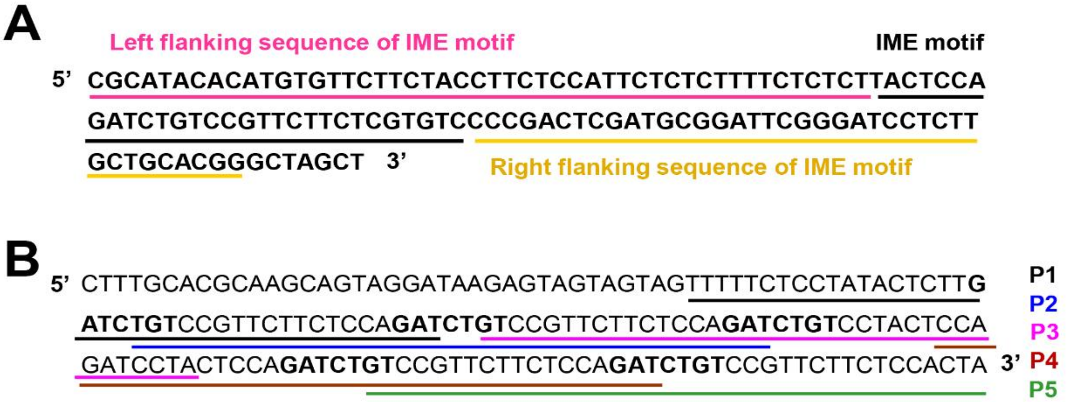

Supplementary Fig. S8 Partial intron-1 sequences of *VRT-A2* used to design theelectrophoretic mobility shift assay probes (Supports Figure 3).

A Design of intron-mediated enhancement (IME), IME-left and IME-right probes based on the IME motif-containing intron-1 fragment shared by *VRT-A2a* and *VRT-A2b* alleles. **B** Design of five IME motif-containing probes, P1 to P5, according to the 157-bp intron-1 fragment specifically found in *VRT-A2b* allele.

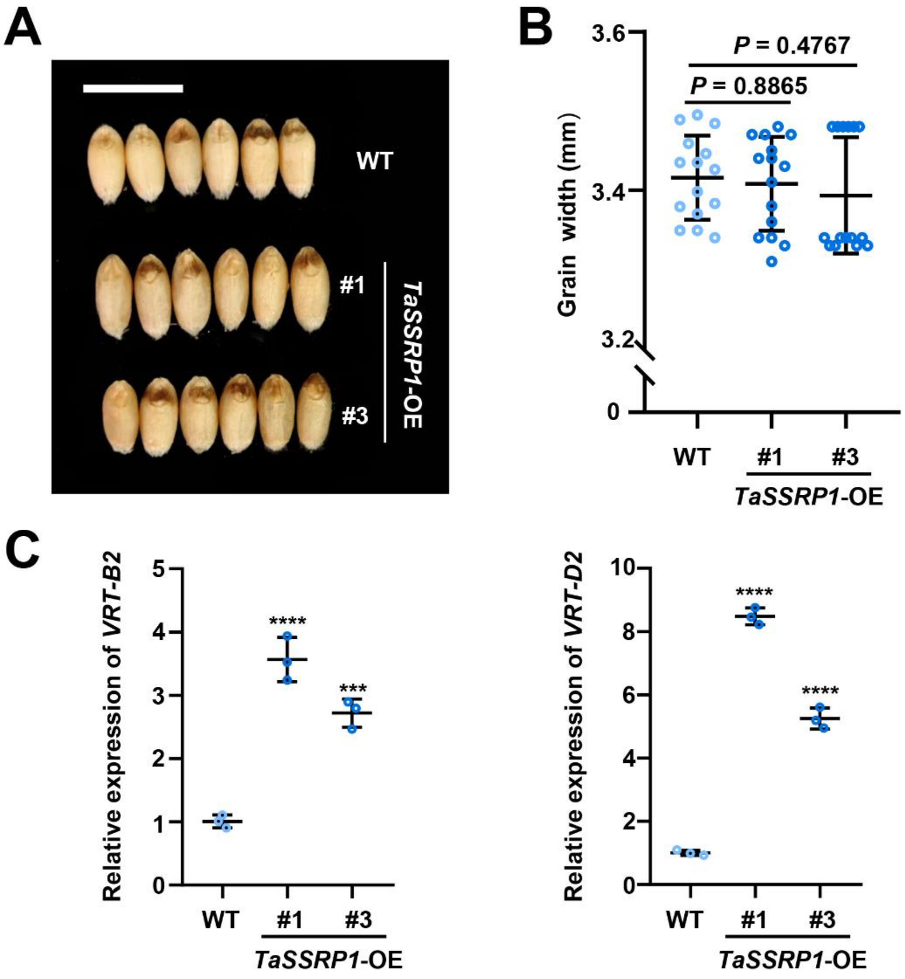

Supplementary Fig. S9 TaSSRP1 does not influence grain width in wheat (Supports Figure 4).

A The grains of wild type (WT) and *TaSSRP1* overexpression (*TaSSRP1*-OE) transgenic plants. Scale bar, 1 cm. The image was digitally extracted for comparison. **B** Statistical analysis of the grain width of WT and *TaSSRP1*-OE. Data are means ± SD (n = 15 independent wheat plants). **C** The expression levels of *VRT-B2* and *VRT-D2* genes in the spikes of WT and *TaSSRP1*-OE transgenic lines. In **B** and **C**, significant difference between WT and *TaSSRP1*-OE was analysed based on one-way ANOVA followed by Dunnett's tests (**** $P < 0.0001$; *** $P < 0.001$).

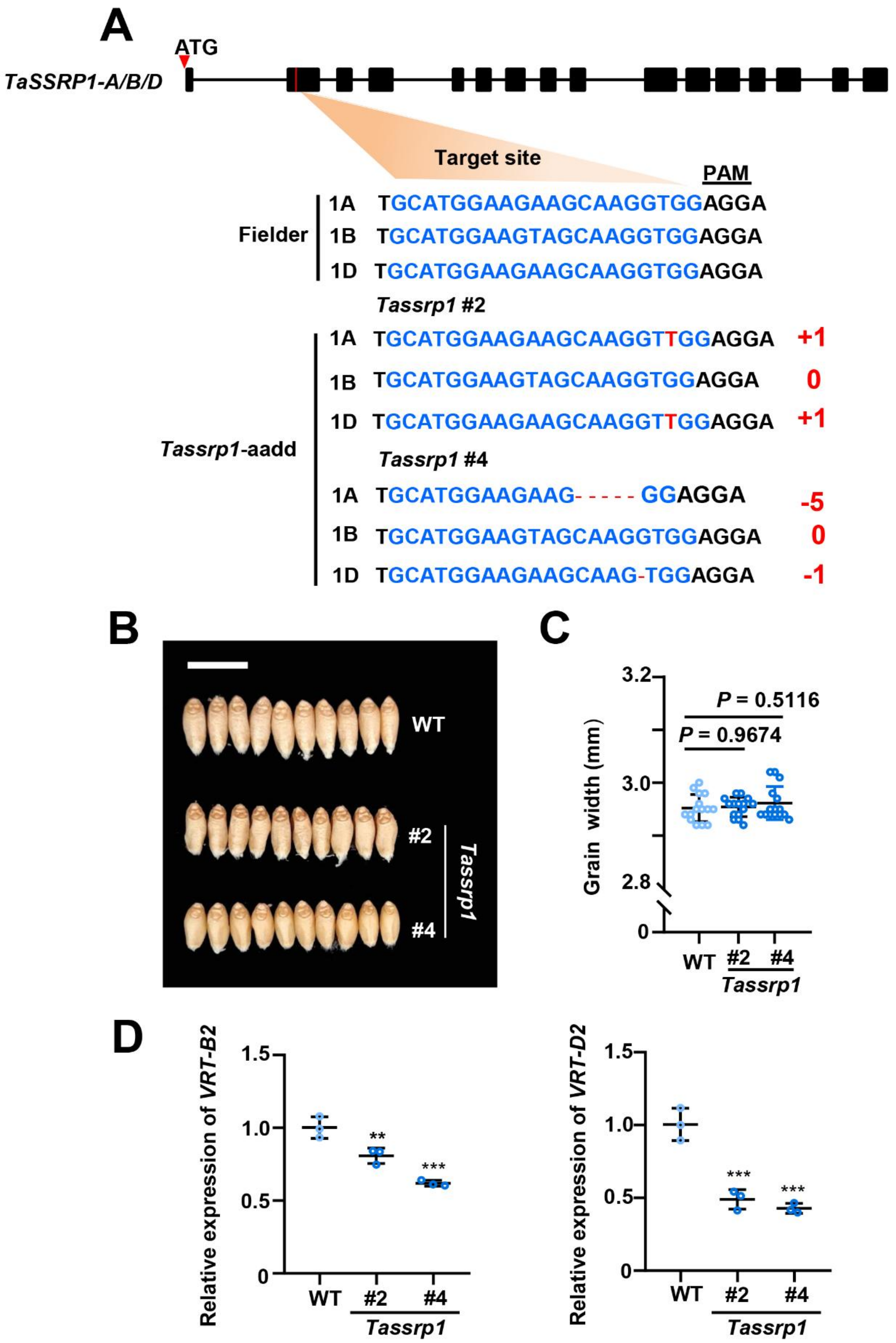

Supplementary Fig. S10 Generation of *Tassrp1* mutant lines and their grain phenotype (Supports Figure 4).

A Mutations in *TaSSRP1-A* and *TaSSRP1-D* genes generated by CRISPR-Cas9 strategy. The gRNA target sites were highlighted in blue and the protospacer adjacent motif (PAM) motifs were directed by black lines. the red dotted lines and letters

represent base deletions and insertions, respectively; the numbers of deletion (−) or insertion (+) bases are shown behind the sequences. **B** Grains of wild type (WT) and the *TaSSRP1-A*- and *TaSSRP1-D*-knocked out *Tassrp1* mutant lines (#2 and #4) shown in (**A**). Scale bar, 1 cm. Images were digitally extracted for comparison**. C** Statistical analysis of the grain width of WT and *Tasspr1* mutant lines. Data are means ± SD (n = 15 independent wheat plants). **D** The expression levels of *VRT-B2* and *VRT-D2* genes in the spikes of WT and *Tasspr1* mutant plants. In **C** and **D**, significant difference between WT and *Tassrp1* was analyzed based on one-way ANOVA followed by Dunnett's tests (*** $P < 0.001$; ** $P < 0.01$).

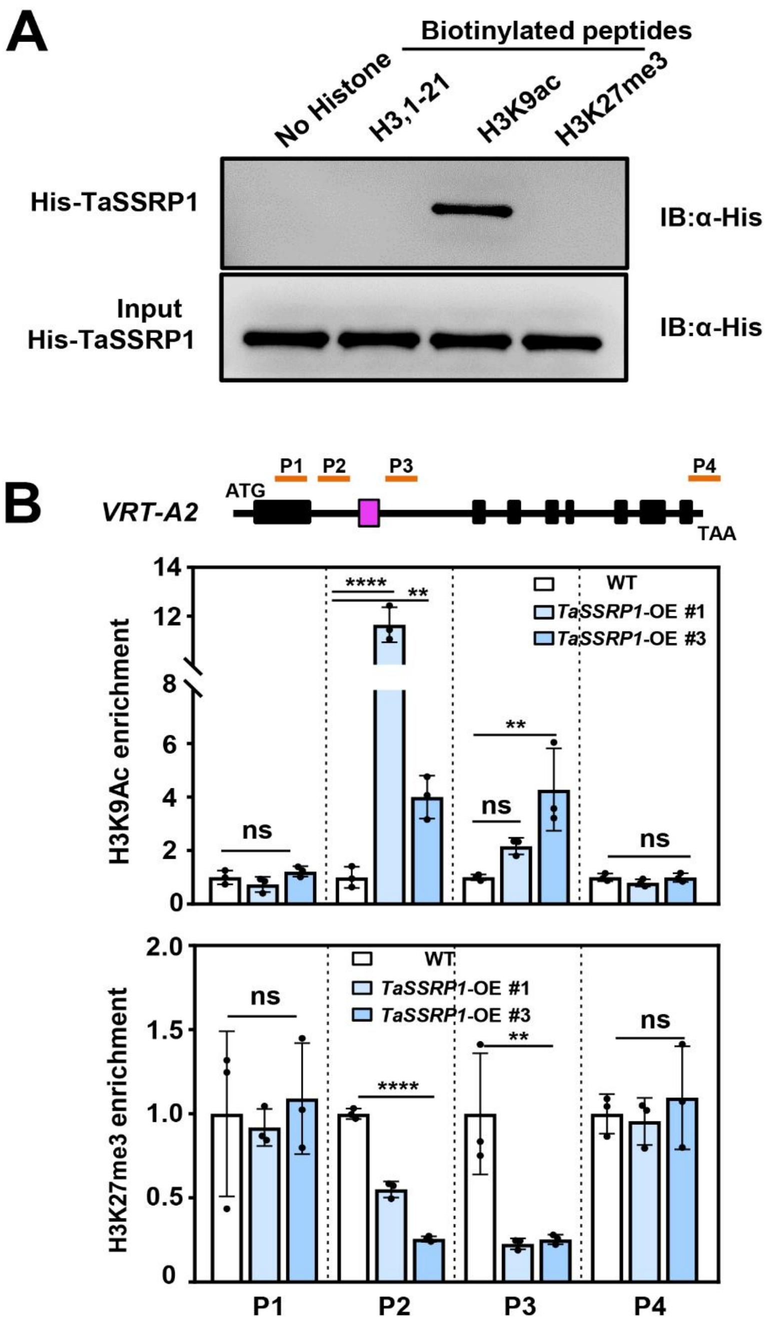

Supplementary Fig. S11 TaSSRP1 influences histone H3 acetylation (H3K9Ac) and H3 trimethylation of lysine 27 (H3K27me3) at the *VRT-A2* locus (Supports Figure 4).

A In vitro pull-down assay by streptavidin beads showing the specific interaction between TaSSRP1 and the H3K9Ac-modified histone. **B** ChIP assays showing the H3K27me3 and H3K9Ac modification levels on the *VRT-A2* gene locus in wild type (WT) and *TaSSRP1* overexpression (*TaSSRP1*-OE) lines. The spikes of WT and *TaSSRP1*-OE with 3-4 cm in length were collected for the ChIP-PCR assay. Data are means ± SD (n = 3 independent biological replications). **** $P < 0.0001$; ** $P < 0.01$; ns, no significant difference (significant difference between WT and *TaSSRP1*-OE based on one-way ANOVA followed by Dunnett's tests). The magenta box indicates the position of the 560-bp *VRT-A2a*-specific fragment in the intron-1 region.

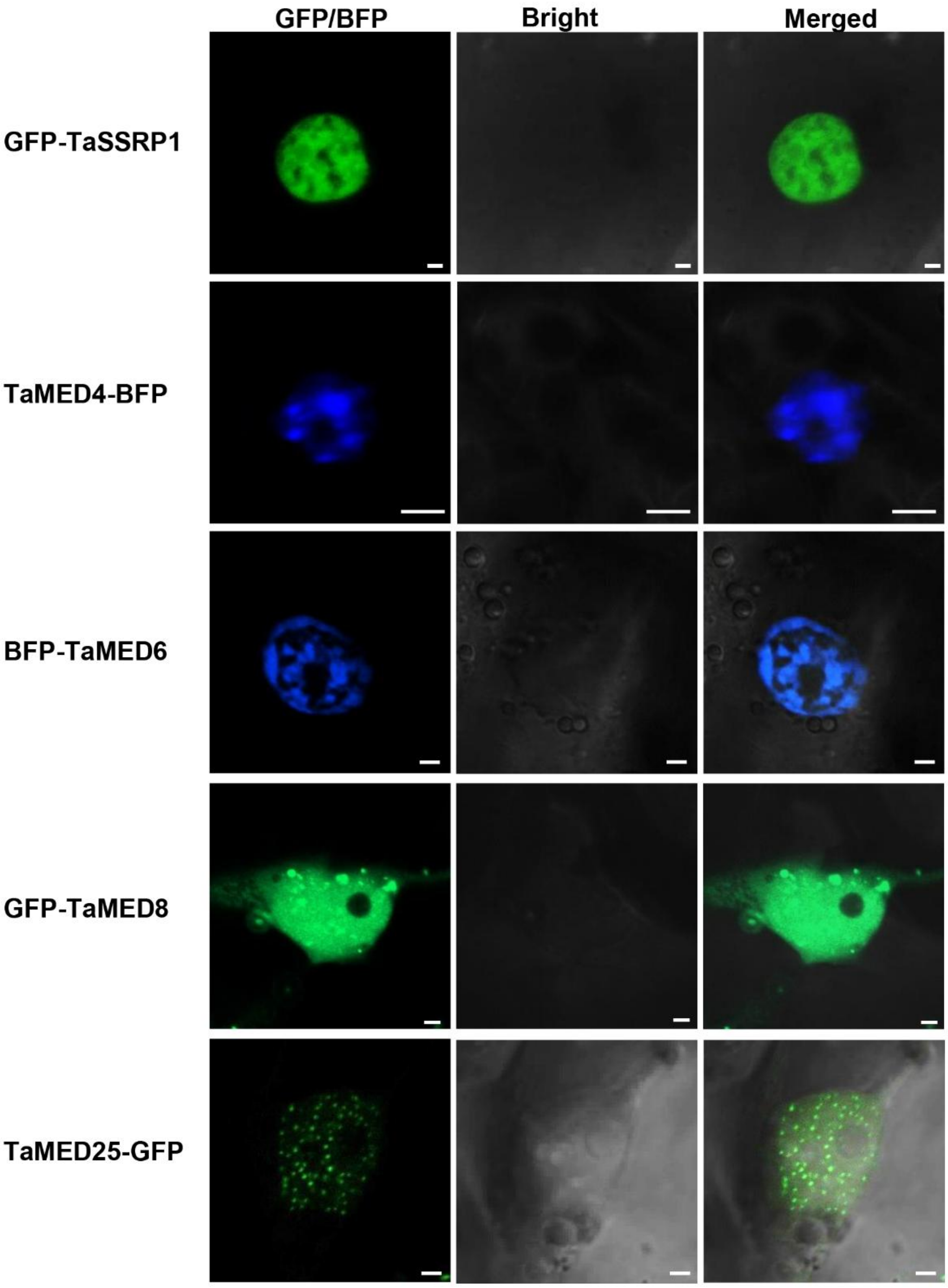

Supplementary Fig. S12 Subcellular localization of the GFP- or BFP-tagged TaSSRP1, TaMED4, TaMED6 and TaMED8 proteins (Supports Figure 5).

Bars = 2 μm.

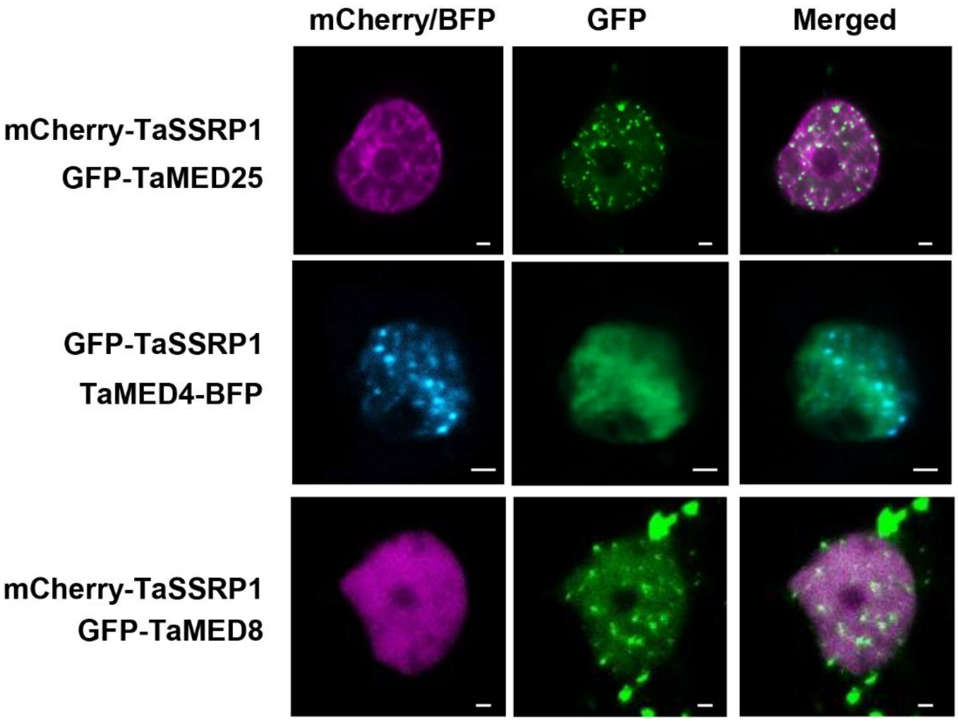

Supplementary Fig. S13 Subcellular co-localization of TaSSRP1 and the Mediator subunits TaMED25, TaMED4 and TaMED8 (Supports Figure 5). Bars = 2 μm.

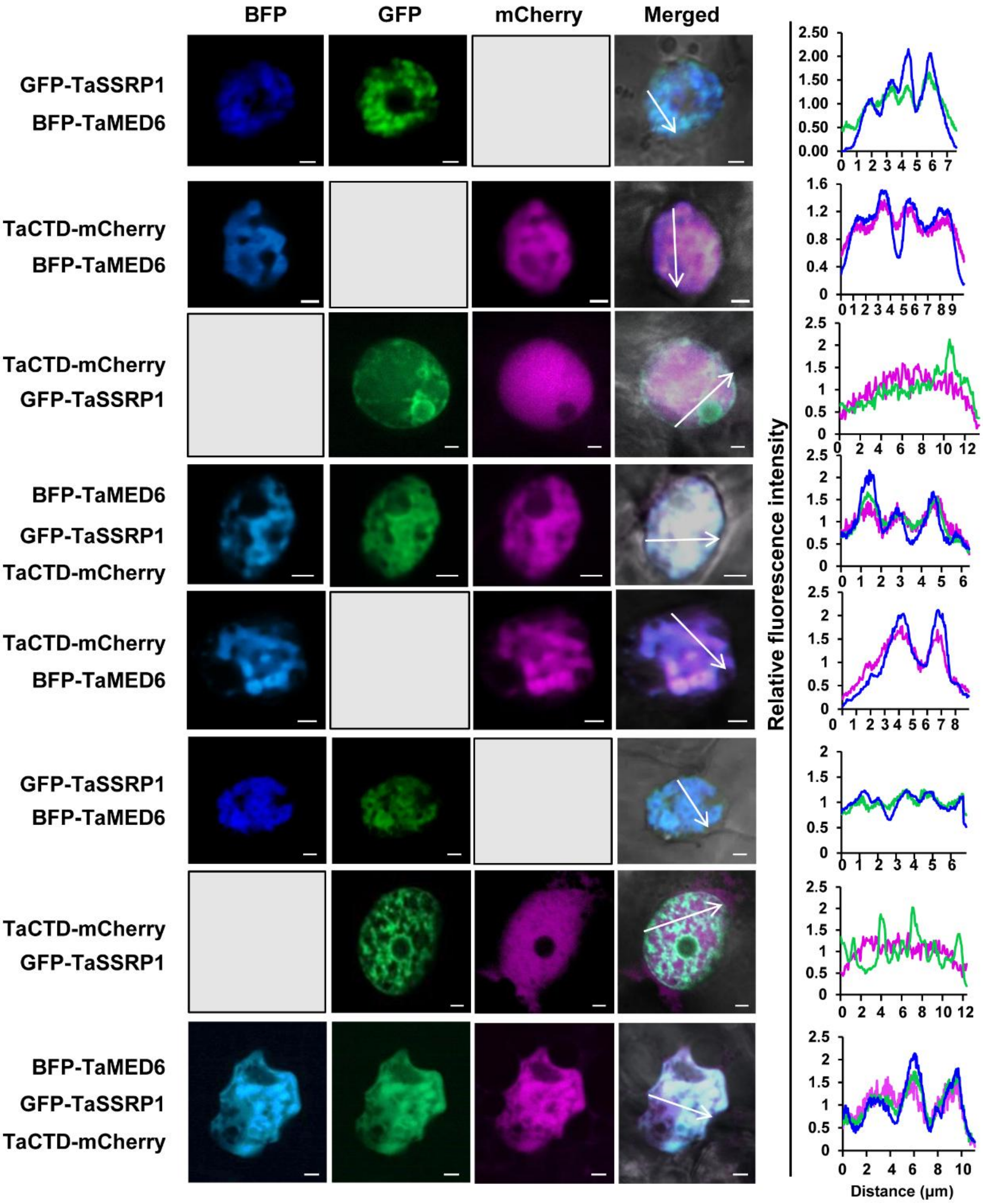

Supplementary Fig. S14 Subcellular co-localization of TaSSRP1, TaMED6 and the C-terminus of wheat RNA Polymerase II (TaCTD) (Supports Figure 5). Confocal microscopy images were shown in the left panels, while relative fluorescence intensities along the white arrows in the merged images were calculated by the Zen software on a Zeiss LSM 880 confocal microscope and showed in the right panels. Bars = 2 μm. The gray color filled boxes illustrate that the corresponding channels were not detected.

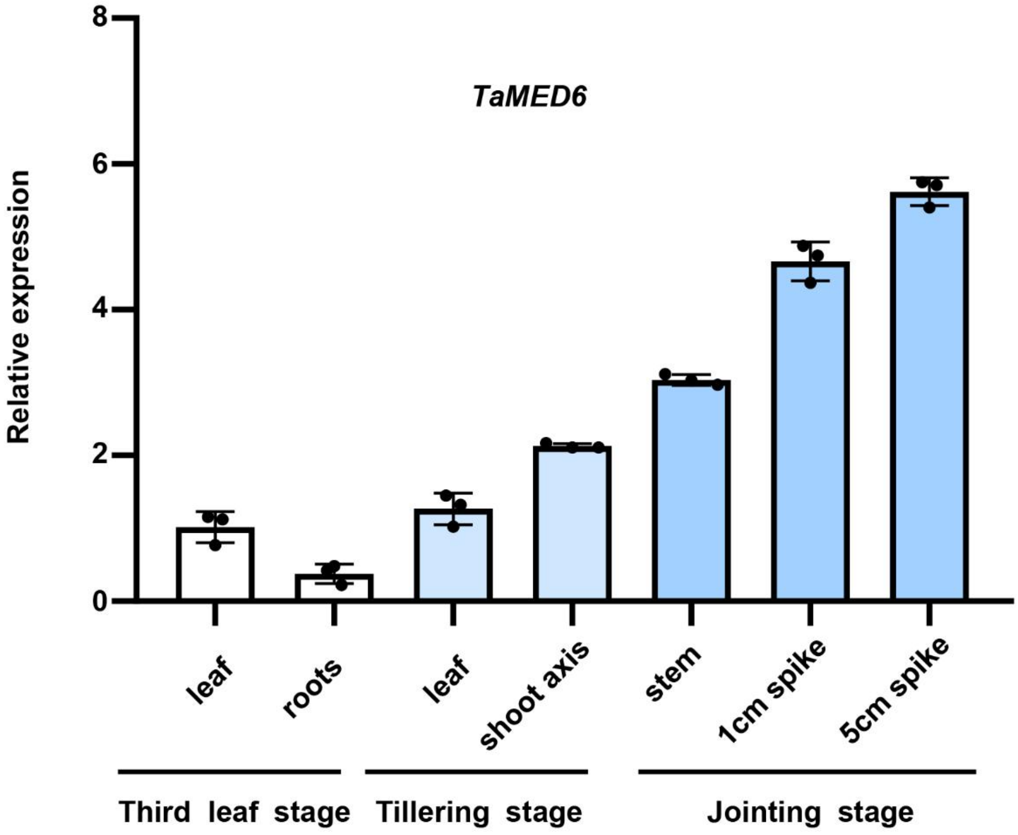

Supplementary Fig. S15 RT-qPCR analysis showing the expression pattern of *TaMED6* in different tissues (Supports Figure 5).

Roots, stems, leaves and spikes were collected from c.v. Fielder. *Actin* was used as an internal control. Different tissues were collected from the pot-grown Fielder in a growth chamber. Data are means ± SD (n = 3 biological independent replicates).

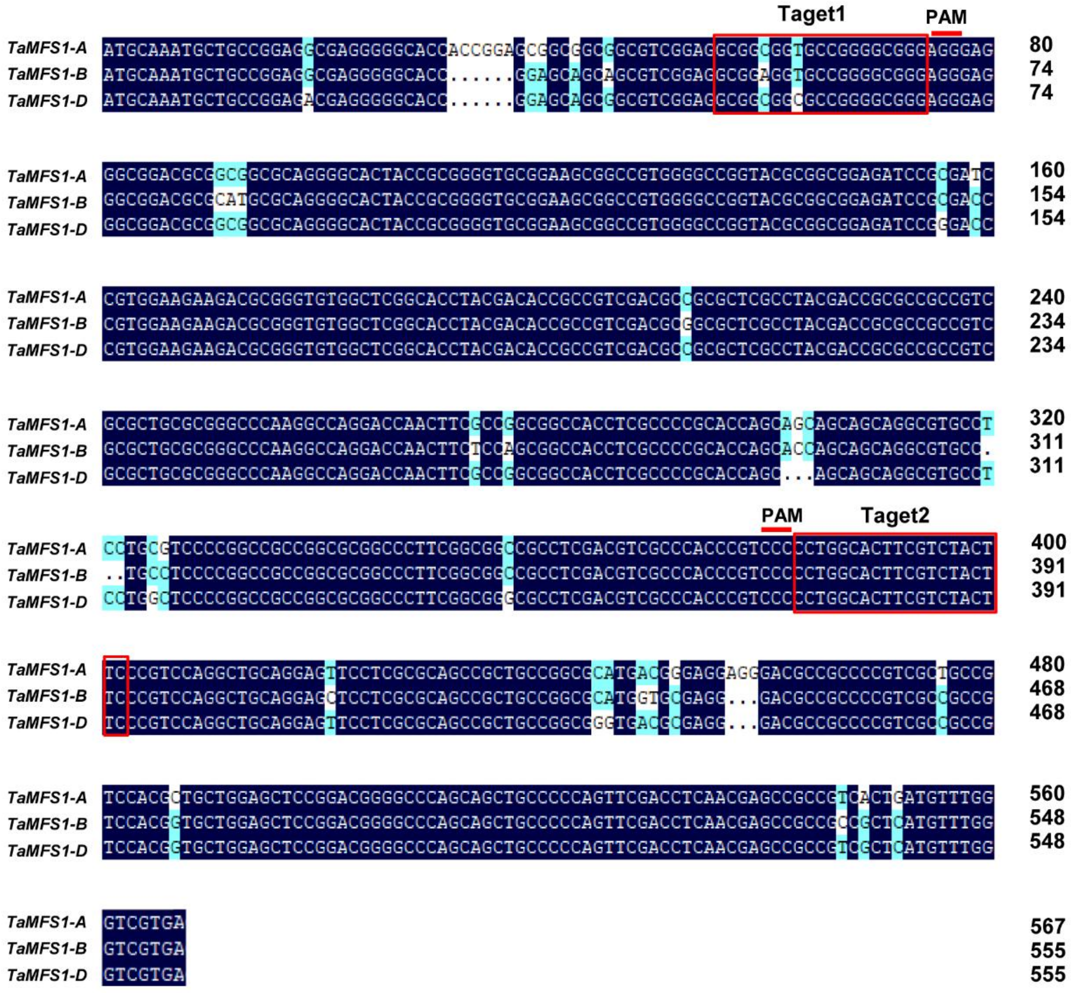

Supplementary Fig. S16 Alignment of the coding sequences of *TaMFS1* homoeologs derived from the A, B and D subgenomes, respectively (Supports Figures 6 and 7).

The two selected target sites (target1 and target2) for CRISPR/Cas9-mediated gene editing are marked by red boxes, and protospacer adjacent motif (PAM) sequences are marked by red lines. The completely conserved (conserved in all three homoeologs) and partially conserved (conserved in two of the three homoeologs) sequences were highlighted in dark blue and cyan blue colors, respectively.

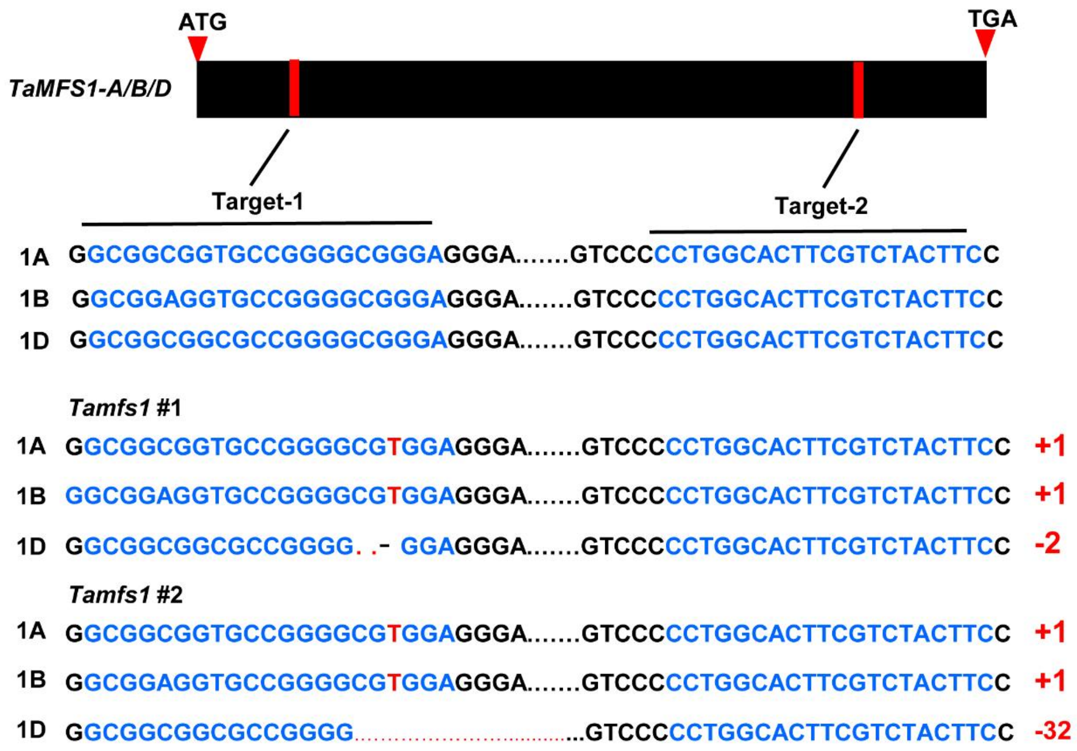

Supplementary Fig. S17 The CRISPR/Cas9-triggered mutations in *TaMFS1-A*, *TaMFS1-B* and *TaMFS1-D* for the generation of *Tamfs1* mutant lines (Supports Figures 6 and 7).

The two selected target sites (target-1 and target-2) for CRISPR/Cas9-mediated gene editing are indicated by black lines; the red dotted lines and letters represent base deletions and insertions, respectively; the numbers of deletion (−) or insertion (+) bases are shown behind the sequences.

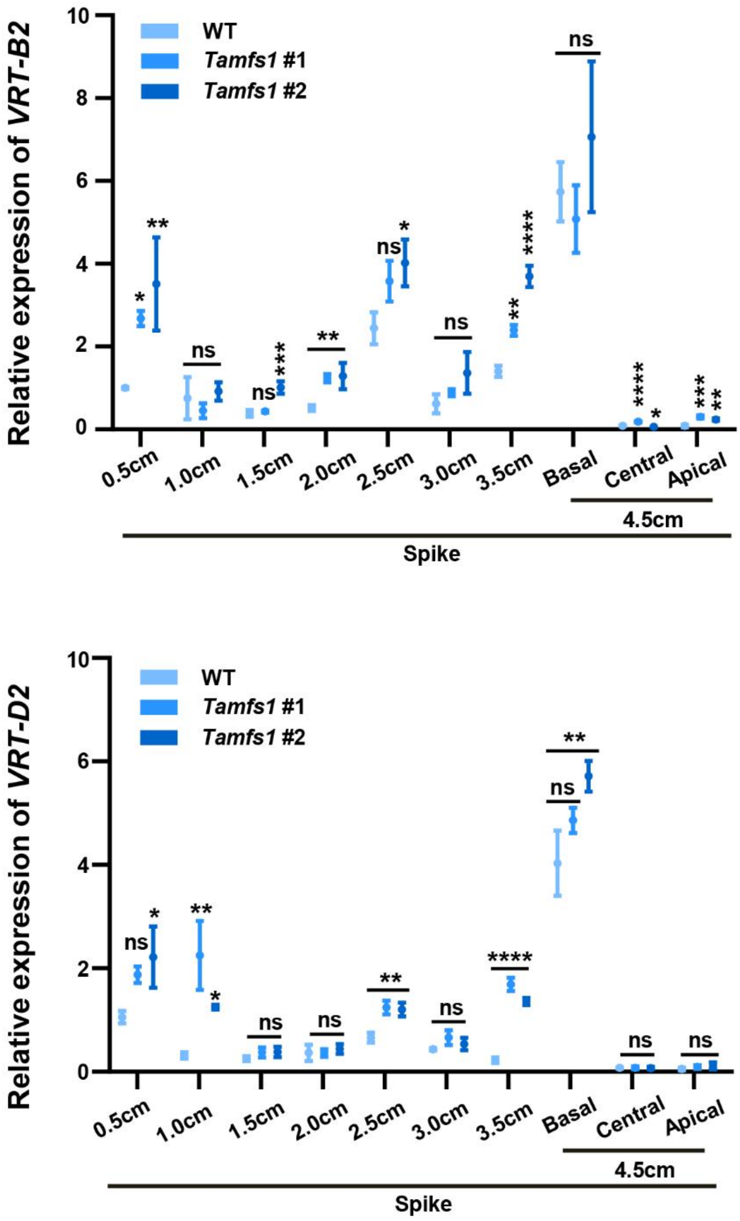

Supplementary Fig. S18 RT-qPCR analysis reveals the expression patterns of *VRT-B2* and *VRT-D2* in spike tissues of different lengths (Supports Figure 6). *Actin* was used as an internal control. Data are means ± SD (n = 3 biological independent replicates). The spikes of wild type (WT) and *Tamfs1* were collected from the plants grown in a growth chamber under long-day photoperiods (16-h-light/8-h-dark, 25 °C/18 °C) at different developmental stages. **** P <0.0001; *** P < 0.001; ** P < 0.01; * P < 0.05 (significant difference between WT and *Tamfs1* based on one-way ANOVA followed by Dunnett's tests). ns, no significant difference.

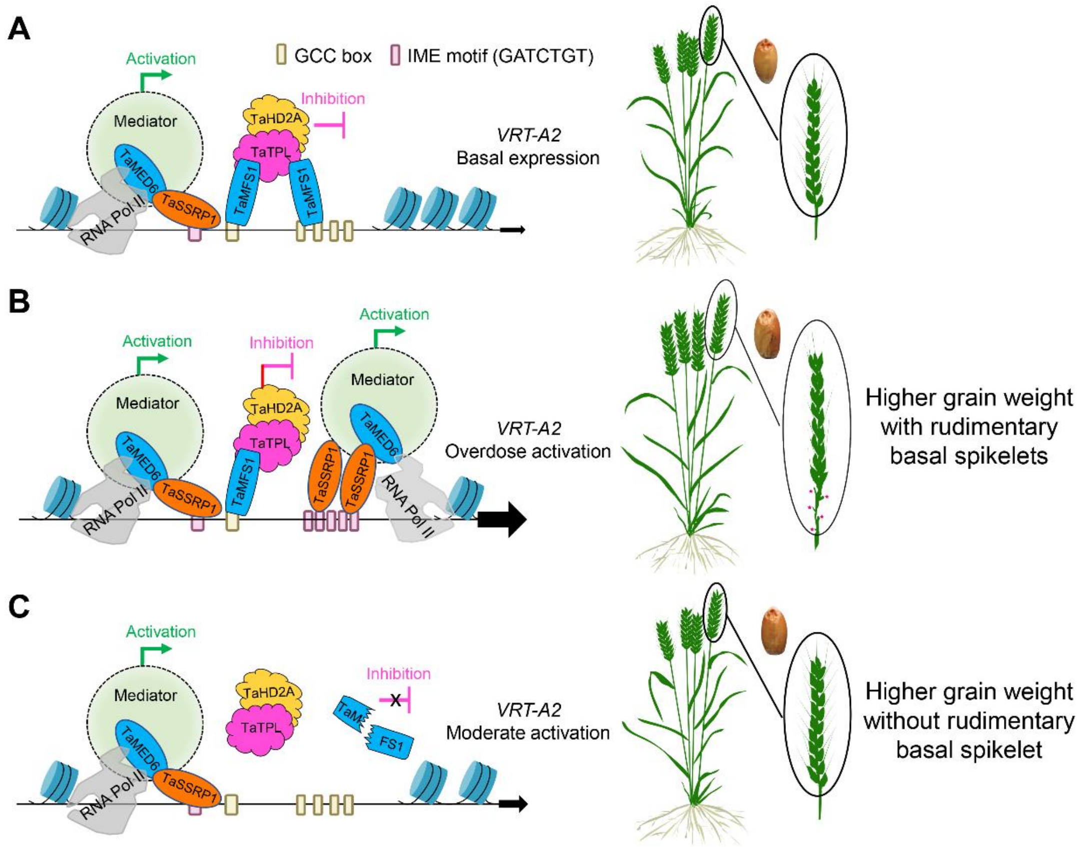

Supplementary Fig. S19 A proposed working model.

A In common wheat with *VRT-A2a* allele, TaMFS1 represses *VRT-A2* expression through binding to the GCC-boxes and recruiting the "TaTPL-TaHD2A" transcriptional repression complex; while TaSSRP1 promotes *VRT-A2* expression via the association with the IME motif and further assembly of "Mediator–RNA Pol II" transcriptional promotion complex. An exquisitely designed "repressor–activator" pair enables a finely controlled basal and low expression of *VRT-A2* in spike and grain tissues. **B** In polish wheat with *VRT-A2b* allele, less GCC-boxes and more IME motifs in the intron-1 region may partially attenuate TaMFS1 binding and TaMFS1-mediated *VRT-A2* repression but enhance TaSSRP1-IME motif interaction and TaSSRP1-triggered *VRT-A2* activation. Although *VRT-A2* activation improves grain weight, it causes increased number of rudimentary basal spikelets and decreased grain number per spike. Such trade-off between spike development and grain weight has dampened the *VRT-A2* effect in increasing grain yield per spike. The red asterisks indicate the rudimentary basal spikelets. **C** By editing *TaMFS1*, the upstream transcriptional repressor of *VRT-A2a*, in common wheat, we enables moderate activation of *VRT-A2* that significantly increases grain weight and grain yield per spike without triggering rudimentary basal spikelets. The expression levels of *VRT-A2* are indicated by the thickness of the black arrows.
