# Supplementary material for: Decoupling the pleiotropic effects of VRT-A2 during reproductive development enhances wheat grain length and weight
Source: Plant Cell. 2025 Feb 14;37(2):koaf024. doi: 10.1093/plcell/koaf024 (PMC11827615; doi:10.1093/plcell/koaf024)
Supplement: koaf024_Supplementary_Data [file tpc.11426.2024-s10.pdf › tpc.11426.2024-s10.pdf]

THE PLANT CELL

Decoupling the pleiotropic effects of VRT-A2 during spike and grain development enhances wheat grain length and weight

Jing Liu, Chaoqun Dong, Xiangqing Liu, Jinquan Guo, Lingling Chai, Weilong Guo, Zhongfu Ni, Qixin Sun, and Jie Liu

Review Timeline:

Submission Date:	18-Oct-2024
Editorial Decision:	20-Dec-2024
Revision Received:	02-Jan-2025
Accepted:	05-Jan-2025

1st Editorial Decision 20-Dec-2024

Dear Dr. Liu:

We have received reviews of your manuscript entitled "Decoupling the pleiotropic effects of VRT-A2 in spike and grain development enhances wheat grain yield." Reviewers have appreciated your manuscript's contribution, leading to a better understanding of VRT-A2 regulation, which enabled the uncoupling of the beneficial grain size increase caused by enhanced expression of VRT-A2 from the other deleterious pleiotropic effects. There are only 2 reviews. However, they are consistent with each other and in line with the editorial consultations. Therefore, on the basis of the advice received, the board of reviewing editors is likely to accept your manuscript for publication in The Plant Cell. This acceptance is contingent on revision based on the comments of our reviewers. In particular, please consider the following:

As per reviewer#2, please change the title which overstates the results when it comes to the effect on yield. As per reviewer#3, please Include expression data on the homoeologous B and D VRT2 genes which might also be relevant to the observed phenotypes. Please follow also all the other comments of both reviewers 2 and 3.

Notes from the Editor-in-Chief: We are trying to make a concerted effort to change green/red comparisons to green/magenta to make our figures understandable to those with color vision deficiencies. We noticed that a small number of your figures utilize red/green contrasts, so as you prepare the final version of the figures, please check the figures for red/green color use. Magenta is a good substitute for red even if the data are labeled as "RFP" or "mCherry" - readers will understand that colors can be changed, and it can be adjusted relatively quickly by using the "hue" setting in Photoshop or similar software. For example, figures 5H and 5I need to be corrected; you might want to check over the Supplemental Figures for similar comparisons. Please note that color changes do not need to be highlighted or tracked in the revised manuscript, but could be noted in the cover letter or response document.

Also, for your bar charts. Stylistically, we prefer solid colors, but we also like modern color schemes that are easy to distinguish and understand. You might read over our figure guide here, with some useful examples:
https://tpc.msubmit.net/html/TPC_Detailed_Figure_Guidelines.pdf

Please highlight all changes and include a detailed annotation of changes in the text, with line numbers, and note your responses to the comments.

To submit your revised manuscript, click:

Link Not Available

If you have any questions about the revision submission procedures, please contact the Editorial Office Staff (tpc-submissions@aspb.org). If you cannot return the revised manuscript within 30 days, please let us know. Otherwise, we will assume that you have elected not to revise the manuscript and withdraw it.

Thank you very much for the privilege of reviewing this work. I look forward to receiving the next version.

Sincerely,

The Plant Cell Editorial Board

Reviewer #2 (Comments for the Author):

The present study by Liu et al. deals with a very interesting and grain yield-relevant phenotype in bread wheat, i.e. improved grain weight.
Here, the remarkable VRT-A2 gene, a pivotal player in the transition from vegetative to reproductive stages in wheat, is well known for its association with traits like long glume and large grain size. This gene has been identified as a subspecies-forming factor in both tetraploid Polish wheat and hexaploid Xinjiang rice wheat. However, activating VRT-A2 boosts grain weight but it also triggers the formation of rudimentary basal spikelets, complicating its use in high-yield wheat breeding due to potential yield penalties.
To tackle this conundrum, authors have uncovered two fascinating regulatory modules that finely tune VRT-A2 expression. First, there's the AP2/ERF-type transcription factor known as multi-floret spikelet1 (TaMFS1), which acts as a gatekeeper, repressing VRT-A2 by enlisting transcriptional co-repressors and histone deacetylases. Then, they discovered the structure-specific recognition protein TaSSRP1, a dynamic activator that directly binds to crucial IME motifs in the intron-1 region of VRT-A2,

energizing its transcriptional activation. TaSSRP1 collaborates with Mediator and RNA polymerase II, co-localizing in the nucleus to create a sophisticated "repressor-activator" duo. In a gripping twist, by knocking out TaMFS1, authors were able to tip the balance in favor of TaSSRP1, leading to a moderate upregulation of VRT-A2 expression. The result? A significant boost in grain weight without the dreaded yield penalty! This innovative strategy not only showcases the potential of manipulating upstream transcriptional regulators but also paves the way for overcoming the challenges posed by pleiotropic genes, potentially enhancing grain weight in wheat.
From my point of view, the current manuscript looks like a thrilling story and shows a deep appreciation for improving the grain weight potential of wheat. The tremendous genetic, molecular and biochemical validation work impresses! I therefore regard this work as being highly interesting and sound; however, a few issues remain unresolved and require reconsideration. Please see my points below:

1) Title: the title unfortunately overstates the results specifically for the "grain yield" achievement. To claim this, authors require many more field experiments under farm-like conditions and planting densities (see also comments by Khaipho-Burch et al. in Nature 2023 Sep;621(7979):470-473 doi: 10.1038/d41586-023-02895-w). I therefore suggest to follow a more modest approach by stating "Decoupling the pleiotropic effects of VRT-A2 during spike and grain development enhances wheat grain length and weight".

2) L556-567; please add plot sizes, row spacing, and planting density (as plants per m2)

Reviewer #3 (Comments for the Author):

This is a comprehensive paper investigating the binding factors of an intron region in VRT2. This is of interest as alleles of VRT2 with variations in this region have been identified to regulate important yield-related traits including glume and seed length. However, these alleles also contain negative phenotypes related to final seed yield. The authors reason that through identifying and understanding the binding partners alleles of these regulators could be used to mediate for the observed negative effects. The manuscript covers a lot of molecular detail regarding protein and DNA interactions and it is very clear to follow the experimental plan of the authors. There are however, a few areas which need to be addressed before publication.

Major comments
1. For consistency in the manuscript the B and D VRT2 genes need to be included as there is an observed phenotype in the CRISPR lines
2. There needs to be clearer justification as to why the same binding assays as used for SSRP were not included for the 3 genes linked to expression repression
3. Please follow the community guidelines on gene and protein names, both in the text and with respect to naming the genes you identify and report in the manuscript. https://link.springer.com/article/10.1007/s00122-023-04253-w
4. Supplementary Figures 2-6 are missing and so cannot be assessed

Minor comments
L146 What about the B and D genome VRT2 expression?
L168. How was the screening for TaMFS1 interactors conducted?
L177. Why these proteins and not the homoeologous genes on the B and D genomes?
L183. You do not show simultaneous interaction. You show that MFS1 has the potential to interact with TPL or HD2A. They may form a complex but that is not supported by your data
L196. A common downstream gene
L287. Why not assess the single CRISPR mutants? Along with the double? Please also include why the triple was not used? Selection issue?
L310 please be more precise which have known locations and which not
L380 why when you remove the repressor of expression do you not see the same ox phenotype? Or why does it not affect the basal spikelets?
Fig1D. please remove the surrounding box as it is obscuring the PI Oe data point and the error bar is not visible.
Fig2B I think the control yeast still shows growth and I would suggest conducting the assay with 3AT as is normal for these assays.
Fig3B-H. By biologically independent are these different leaves of the same plant? I think this might be technical replication. Does the data show different plant leaves responses?

Please also note the following:

-The Plant Cell now requires authors to complete and submit an author revisions checklist upon submission of a revised

manuscript. The aim of the checklist is to aid authors in preparing a high-quality manuscript, facilitate the review and assessment of revised manuscripts, and help to ensure that journal standards are maintained across the board. If your manuscript is accepted, the completed checklist will be published as supplemental material attached to the article online. Please download a copy of the checklist (pdf fillable form) at this link, for submission with your revised manuscript: https://tpc.msubmit.net/html/Author_Revisions_Checklist.pdf.

-Supplemental materials should be restricted to large datasets and tables, presentation of replicates, and validation of reagents, methods, or genotypes. Any data that are used to support the major claims must be in the main manuscript. Supplemental figure legends must indicate what figure in the main manuscript is supported by the supplemental data presented. Please justify how each of the supplemental figures meet the criteria.

-Sampling methods and the nature of "biological replicates" should be described precisely (i.e. different plants, parts of plants, pooled tissue, independent pools of tissue, sampled at different times, etc.), along with a clear description of and rationale for any statistical analyses conducted. The reader should know exactly what was sampled; what forms the basis of the calculation of any means and statistical parameters reported. This is also necessary to ensure that proper statistical analysis was conducted.

TPC2024-RA-11426

Point-by-point responses to the Reviewers' comments

Reviewer #2 (Comments for the Author):

1. The present study by Liu et al. deals with a very interesting and grain yield-relevant phenotype in bread wheat, i.e. improved grain weight. Here, the remarkable *VRT-A2* gene, a pivotal player in the transition from vegetative to reproductive stages in wheat, is well known for its association with traits like long glume and large grain size. This gene has been identified as a subspecies-forming factor in both tetraploid Polish wheat and hexaploid Xinjiang rice wheat. However, activating *VRT-A2* boosts grain weight but it also triggers the formation of rudimentary basal spikelets, complicating its use in high-yield wheat breeding due to potential yield penalties. To tackle this conundrum, authors have uncovered two fascinating regulatory modules that finely tune *VRT-A2* expression. First, there's the AP2/ERF-type transcription factor known as multi-floret spikelet1 (TaMFS1), which acts as a gatekeeper, repressing *VRT-A2* by enlisting transcriptional co-repressors and histone deacetylases. Then, they discovered the structure-specific recognition protein TaSSRP1, a dynamic activator that directly binds to crucial IME motifs in the intron-1 region of *VRT-A2*, energizing its transcriptional activation. TaSSRP1 collaborates with Mediator and RNA polymerase II, co-localizing in the nucleus to create a sophisticated "repressor-activator" duo. In a gripping twist, by knocking out TaMFS1, authors were able to tip the balance in favor of TaSSRP1, leading to a moderate upregulation of *VRT-A2* expression. The result? A significant boost in grain weight without the dreaded yield penalty! This innovative strategy not only showcases the potential of manipulating upstream transcriptional regulators but also paves the way for overcoming the challenges posed by pleiotropic genes, potentially enhancing grain weight in wheat. From my point of view, the current manuscript looks like a thrilling story and shows a deep appreciation for improving the grain weight potential of wheat. The tremendous genetic, molecular and biochemical validation work impresses! I therefore regard this work as being highly interesting and sound.

Response:

We appreciate for your positive comments and your assistance in refining our work. We have made revisions to enhance the quality of our manuscript according to your suggestions.

2. However, a few issues remain unresolved and require reconsideration. Please see my points below:

1) Title: the title unfortunately overstates the results specifically for the "grain yield" achievement. To claim this, authors require many more field experiments under farm-like conditions and planting densities (see also comments by Khaipho-Burch et al. in Nature 2023 Sep;621(7979):470-473 doi: 10.1038/d41586-023-02895-w). I therefore suggest to follow a more modest approach by stating "Decoupling the pleiotropic effects of VRT-A2 during spike and grain development enhances wheat grain length and weight".

Response: Thank you very much for these valuable suggestions. In this version, we revised the title as "Decoupling the pleiotropic effects of *VRT-A2* during spike and grain development enhances wheat grain length and weight". We also revised other parts of the manuscript to avoid the misleading description about grain yield.

2) L556-567; please add plot sizes, row spacing, and planting density (as plants per m^2)

Response: Thanks for the suggestion. In each replication, wheat seeds were hand planted in 5-row plots of 1.5 m in length spaced 0.30 m apart with 20 seeds per row. In the Methods section, we included information about plot sizes and row spacing in "plant materials and growth conditions".

Reviewer #3 (Comments for the Author):

This is a comprehensive paper investigating the binding factors of an intron region in *VRT2*. This is of interest as alleles of *VRT2* with variations in this region have been identified to regulate important yield-related traits including glume and seed length. However, these alleles also contain negative phenotypes related to final seed yield.

The authors reason that through identifying and understanding the binding partners alleles of these regulators could be used to mediate for the observed negative effects. The manuscript covers a lot of molecular detail regarding protein and DNA interactions and it is very clear to follow the experimental plan of the authors. There are however, a few areas which need to be addressed before publication.

Response:

We appreciate for your insightful comments and your valuable suggestions in refining our work.

Major comments:

1. For consistency in the manuscript the B and D *VRT2* genes need to be included as there is an observed phenotype in the CRISPR lines.

Response: Thanks for these valuable suggestions. In this version, we conducted qRT-PCR analyses to compare the expression levels of *VRT-B2* and *VRT-D2* in the spikes of *TaMFS1*-OE, *TaSSRP1*-OE and *Tassrp1* lines. Results showed that the expression levels of *VRT-B2* and *VRT-D2* were indeed suppressed in the *TaMFS1*-OE material (Supplementary Fig. 1C). Similarly, *VRT2* expression was positively regulated by TaSSRP1. The expression of *VRT-B2* and *VRT-D2* was significantly increased in *TaSSRP1* overexpression materials as compared to the wild type; while in *Tassrp* mutant lines, the expression of *VRT-B2* and *VRT-D2* was inhibited (Supplementary Figs. 9C and 10D).

2. There needs to be clearer justification as to why the same binding assays as used for SSRP were not included for the 3 genes linked to expression repression.

Response: In our previous study, we have performed such binding assays and confirmed that TaMFS1 could directly bind to the GCC-box cis-elements located in the intron-1 region of *VRT-A2*; while mutations in the GCC-box cis-elements attenuated such binding (Liu *et al.*, 2021). Therefore, in this manuscript, we did not include the binding assay results.

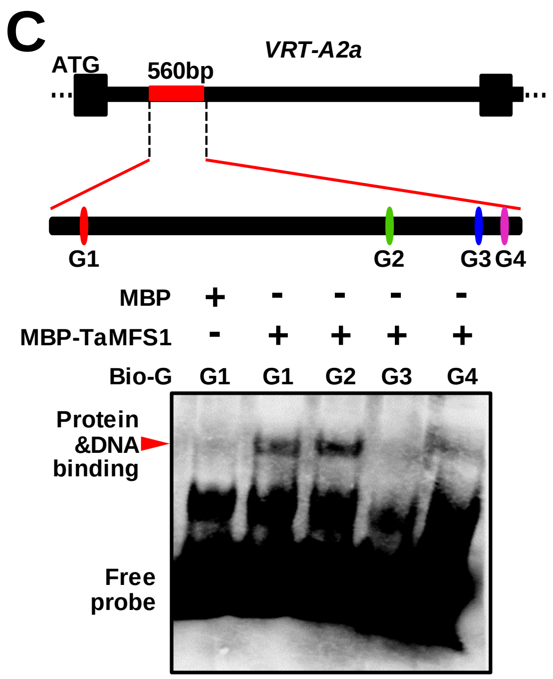

Figure 5C (Liu et al., 2021)

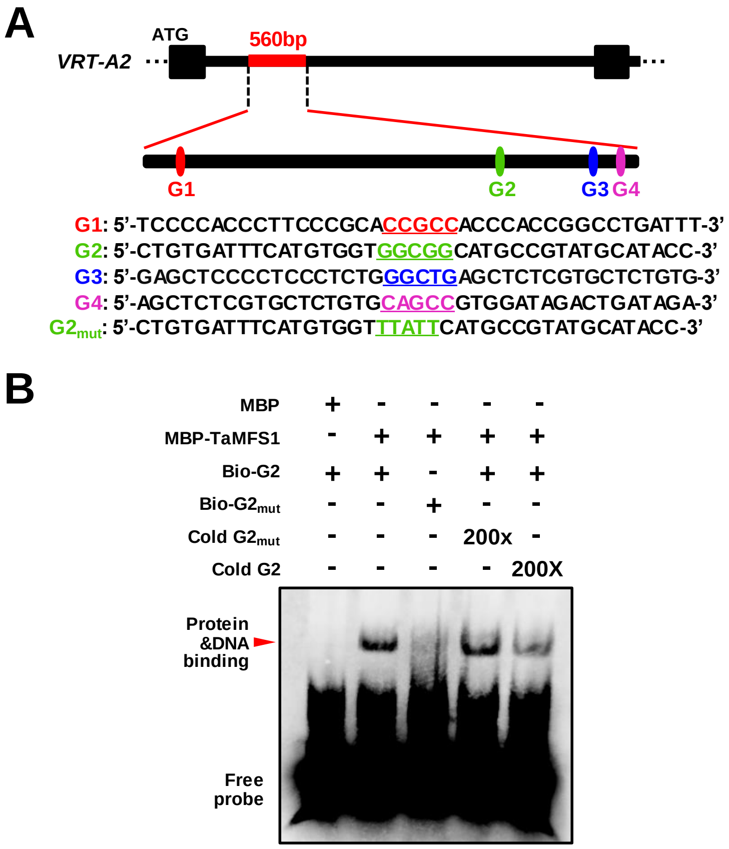

Supplemental Figure 16 (Liu *et al.*, 2021)

3. Please follow the community guidelines on gene and protein names, both in the text and with respect to naming the genes you identify and report in the manuscript. https://link.springer.com/article/10.1007/s00122-023-04253-w.

Response: Thank the reviewer for this comment. In this version, we checked all gene names in the manuscript, and revised their names properly according the community guidelines on gene and protein names.

4. Supplementary Figures 2-6 are missing and so cannot be assessed.

Response: Sorry for missing these figures. In this version, we added the figures in the supplementary information.

Minor comments

L146 What about the B and D genome *VRT2* expression?

Response: In this version, we conducted qRT-PCR analyses to compare the expression levels of *VRT-B2* and *VRT-D2* in the spikes of *TaMFS1*-OE. Please see the results shown in Supplementary Fig. 1C.

L168. How was the screening for TaMFS1 interactors conducted?

Response: Sorry for missing the information. We screened TaMFS1 interactors with yeast two-hybrid. We added this description in the main text.

L177. Why these proteins and not the homoeologous genes on the B and D genomes?

Response: Based on protein sequence alignment and phylogenetic tree construction analysis (Supplementary Fig. 2), the homoeologous proteins derived from different subgenomes (A, B and D) show extremely high-level sequence identities. Therefore, in this study, we just used the A subgenome-derived homologous proteins of TaHDA1-A (encoded by TraesCS7A02G365600) and TaHDA6-A (encoded by TraesCS6A02G181100) as representatives for interaction analyses.

L183. You do not show simultaneous interaction. You show that MFS1 has the

potential to interact with TPL or HD2A. They may form a complex but that is not supported by your data

Response: Thank the reviewer for this comment. In this version, we revised it as "given that TaMFS1 interacts with both TaTPL and TaHD2A...".

L196. A common downstream gene

Response: We revised it according to the suggestion.

L287. Why not assess the single CRISPR mutants? Along with the double? Please also include why the triple was not used? Selection issue?

Response: Thank the reviewer for this comment. We know wheat contains three homologous groups of ABD, so there are three copies of the same gene, and usually the functions of the three genes are relatively conserved. When one copy is knocked out, the other copies with function may supplement the function. Therefore, when studying wheat gene function by reverse genetics, triple mutants are usually used as research materials. In this study, we made efforts to obtain the triple mutants, but only detected double mutant lines with the A and D copies of the homoeologs being simultaneously knocked out, possibly due to the reasons: 1) the numbers of T_0 lines were limited; 2) the region including the B subgenome copy of TaSSRP1 may have complex structure that is resistant to the Cas9-mediated editing.

In our future works, we may generate single and triple mutants by generating more T_0 editing lines, which may help to dissect the functional diversities among the three homoeologs of TaSSRP1.

L310 please be more precise which have known locations and which not

Response: According to the suggestion, in this version, we added the information about the locations of these Mediator subunits.

L380 why when you remove the repressor of expression do you not see the same ox phenotype? Or why does it not affect the basal spikelets?

Response: It is known that plants are complex organisms. The expression of a gene is normally regulated by multiple components. Although we confirmed that TaMFS1 is located upstream of *VRT-A2* to regulate its expression. However, we believe that in addition to TaMFS1, there should be multiple regulators (either positive or negative ones) that simultaneously regulate the expression of *VRT-A2*. Thus, we assumed that editing *TaMFS1* may have relatively weaker biological effects than directly overexpressing *VRT-A2* in activating the expression of *VRT-A2* (Fig. 6C). This may explain the less pleotropic effect of *TaMFS1* editing than *VRT-A2* overexpression in triggering the increase of rudimentary basal spikelets.

Fig 1D. please remove the surrounding box as it is obscuring the PI Oe data point and the error bar is not visible.

Response: We removed the surrounding boxes according to the suggestion. Please see Figs. 1D, 2M and 4G.

Fig 2B I think the control yeast still shows growth and I would suggest conducting the assay with 3AT as is normal for these assays.

Response: In Fig. 2B, the growth rates of the negative controls were obviously lower than the TaTPL/TaMFS1 co-expression samples, although they showed weak growth at the highest concentration. We believe that our current results support the positive and strong interaction between TaTPL and TaMFS1, when comparing TaTPL/TaMFS1 co-expression samples with the negative controls.

Fig 3B-H. By biologically independent are these different leaves of the same plant? I think this might be technical replication. Does the data show different plant leaves responses?

Response: Considering that the growth status of tobacco plants may affect the results, we always choose leaves from independent tobacco plants for analyses, aiming to ensure the reliability of the results, and then perform statistical analysis. In each assay, we used at least nine biologically independent *N. benthamiana* leaves collected from

nine independent plants for statistical analysis. Please also see the description in the "Firefly luciferase (LUC) complementation imaging (LCI) assay" part in Methods section.

References

Liu J, et al. Ectopic expression of *VRT-A2* underlies the origin of *Triticum polonicum* and *Triticum petropavlovskyi* with long outer glumes and grains. Mol. Plant 14, 1472-1488 (2021).

Dear Dr. Liu:

Thanks for addressing all the reviewers' comments. We are pleased to inform you that your paper entitled "Decoupling the pleiotropic effects of VRT-A2 during spike and grain development enhances wheat grain length and weight" has been accepted for publication in The Plant Cell, pending a final editorial review by a science editor. At this stage, your manuscript will be evaluated by a science editor with respect to its presentation of scientific content, compliance with journal policies, and presentation for a broad readership. The Plant Cell has contracted with Plant Editors (planteditors.com) to provide this service to our authors, and you will soon receive additional information on this process.

Please note that each author needs to link their ORCID identifier to their account in the system before your manuscript can be published. If any authors do not have an ORCID linked to their account, they will receive a message with a link to complete this task. Please ensure that ALL of your coauthors have completed this task as soon as possible.

Shortly after your paper is sent to production, you will receive an email from our publisher's author support team, **SciPris**, with information on article processing charges, as well as the types of Open Access license you would be able to purchase. The accepted version of your manuscript will not appear on our Advance Articles page until the license is selected and signed, so please look out for an email from SciPris. For more information see the Article Publication Fees section of our author guidelines: https://academic.oup.com/plcell/pages/General-Instructions

The Plant Cell and The Arabidopsis Information Resource (TAIR) are collaborating to collect functional annotation data about Arabidopsis genes from authors. This includes information about the gene's molecular function (e.g., kinase activity, ATP synthetase activity), the biological process/es it is involved in (e.g., endosperm development, threonine biosynthesis), its subcellular location (e.g., nucleus, ER), anatomical or developmental expression pattern (e.g., leaf, ovule, flower stage 10, seedling stage), or its partner in a protein-protein interaction (e.g., AT1G01010 interacts with AT1G01020).

If your paper contains results falling into one or more of these categories for Arabidopsis genes, we request that you now submit these data for inclusion in TAIR by filling in the form provided at the following URL: https://www.arabidopsis.org/doc/submit/functional_annotation/123. If you need further clarification on what types of data can be submitted please contact curator@arabidopsis.org.

Finally, we encourage your submission of artwork for the journal cover. If there is an image or illustration related to your paper that you would like to have considered, please email it in .pdf or .tif format to clowe@aspb.org. An image title, brief, 2-3 sentence description of the image, and the name of the person credited for the image are also encouraged. If your image is selected to appear on the online cover of the journal, you will be contacted with the scheduled issue date and further information.

We look forward to seeing your paper published.

Sincerely,

The Plant Cell Board of Editors

=====
IMPORTANT REMINDER: PEER REVIEW REPORTS
=====

If you opted to publish a peer review report along with your article during the original submission process, it will be prepared by the editorial staff and publicly posted with your manuscript, inside the zip file that contains any other supplemental material. As a reminder, the peer review report is a public record of all comments from editors and reviewers, as well as your prior responses, as you received them in the decision letters for each draft of your manuscript. If you agreed to publish this report and have changed your mind, or are not sure if you selected this option, please contact the editorial office as soon as possible before signing the license agreement from our publisher.

=====
REVIEWER COMMENTS
=====

----FOR ASPB OFFICE USE ONLY (DO NOT EDIT)----

MSID: 41855
Corresponding Author: Prof. Dr. Liu
